# Ultra-dense SNP genetic map construction and identification of *SiDt* gene controlling the determinate growth habit in *Sesamum indicum* L.

**DOI:** 10.1038/srep31556

**Published:** 2016-08-16

**Authors:** Haiyang Zhang, Hongmei Miao, Chun Li, Libin Wei, Yinghui Duan, Qin Ma, Jingjing Kong, Fangfang Xu, Shuxian Chang

**Affiliations:** 1 Henan Sesame Research Center, Henan Academy of Agricultural Sciences, Zhengzhou, Henan, 450002, People’s Republic of China

## Abstract

Sesame (*Sesamum indicum* L.) is an important oilseed crop and has an indeterminate growth habit. Here we resequenced the genomes of the parents and 120 progeny of an F_2_ population derived from crossing Yuzhi 11 (indeterminate, *Dt*) and Yuzhi DS899 (determinate, *dt1*), and constructed an ultra-dense SNP map for sesame comprised of 3,041 bins including 30,193 SNPs in 13 linkage groups (LGs) with an average marker density of 0.10 cM. Results indicated that the same recessive gene controls the determinacy trait in *dt1* and a second determinate line, *dt2* (08TP092). The QDt1 locus for the determinacy trait was located in the 18.0 cM–19.2 cM interval of LG8. The target SNP, *SiDt*27-1, and the determinacy gene, DS899s00170.023 (named here as *SiDt*), were identified in Scaffold 00170 of the Yuzhi 11 reference genome, based on genetic mapping and genomic association analysis. Unlike the G397A SNP change in the *dt1* genotype, the *SiDt* allele in *dt2* line was lost from the genome. This example of map-based gene cloning in sesame provides proof-of-concept of the utility of ultra-dense SNP maps for accurate genome research in sesame.

Sesame (*Sesamum indicum* L., 2n = 2x = 26) belongs to the Pedaliaceae genus and is an ancient oil seed crop^1^. Sesame seed has a high oil and antioxidant (such as lignan) content and is regarded as the ‘Queen of the oil seeds’^2^. At present, sesame is planted mainly in tropical and subtropical regions of Asia, Africa and Southern America, and had a total harvest area of 8.10 million hectares and an average annual production of 4.67 million tons from 2010-2014 (FAO data). India, Myanmar, Sudan and China are the four top countries for sesame production.

In angiosperms, floral induction initiates the transition of vegetative shoot meristems to inflorescence meristems. In indeterminate annual plants, floral formation continues throughout the whole reproductive life, giving rise to indeterminate inflorescences. Determinate plants, on the other hand, can form a terminal flower cluster, as the shoot apical meristem is converted into a terminal floral meristem^3^,^4^. Sesame is an indeterminate crop and has a long flowering stage of greater than one month. Its indeterminate growth habit results in nonsynchronous ripening of capsules, which, together with capsule shattering, leads to poor adaptation to mechanical harvesting^5^,^6^,^7^. A determinate mutant named Yuzhi DS899 (*dt1* line) was created from line 91–0 of the sesame cultivar Yuzhi 11 (selected as the reference genome for the Sesame Genome Project) using ethylmethane sulfonate (EMS) mutagenesis in 2009 (China patent application No.: ZL2015108760163). The *dt1* mutant line has a shorter flowering period and develops 8–20 capsule nodes. The seed ripens concurrently, making it more suitable for mechanical harvesting. An additional determinacy genotype, 08TP092 (*dt2* line), has been induced by gamma ray (5,000 Grey) mutagenesis^8^. The determinacy habit in the *dt2* type limits the number of capsule nodes and this number is not affected by environmental factors^3^,^6^. The molecular mechanism and associated molecular genetic information underlying these two phenotypes has not yet been systematically explored.

A genetic linkage map is a prerequisite for better understanding the inheritance of traits on a genome-wide level^9^. Fine mapping of quantitative trait loci (QTL) and candidate genes related to specific traits are traditionally performed using high resolution genetic linkage maps^10^. In recent years, high throughput next-generation sequencing technology (NGS) has been used for constructing high-density or ultra-dense SNP (single nucleotide polymorphism) genetic maps in crops and enhances genome assembly and map-based gene identification^10^,^11^,^12^,^13^. The first genetic linkage map for sesame was constructed in 2009, with 220 AFLP (amplified fragment length polymorphism) and RSAMPL ((Random Selective Amplification of Microsatellite Polymorphic Loci) markers in 30 linkage groups (LGs) spanning a total length of 936.72 cM^14^. Subsequently, densification of the genetic map to carry 653 SSR (simple sequence repeats), AFLP and RSAMPL markers was performed in 2013 and facilitated the location of four QTLs linked to sesame seed coat color^15^. In recent years, with the aid of reduced representation genome sequencing (RRGS) and NGS techniques, three sesame SNP genetic linkage maps have been constructed^16^,^17^,^18^. In the SNP map constructed using SLAF-seq (Specific Length Amplified Fragment sequencing) (herein named ‘SLAF map’), 1,079 (87.51%) of 1,233 SNPs were mapped onto 15 LGs with a total length of 1,474.87 cM^16^. This SNP map was subsequently improved by adding new RAD-seq (Restriction-site Associated DNA sequencing) data and used for QTL mapping of plant height and seed coat color traits in 2016^18^. In this ‘improved SNP map’, 1,522 bins are distributed on 13 LGs with a total length of 1,090.99 cM. The SNP map constructed using RAD-seq in 2014 (abbreviated here as ‘RAD map’) was comprised of 1,230 markers in 14 LGs over a total length of 844.46 cM^17^. All five maps are far from saturation with fine-scaled markers, and thus provide limited support for sesame genome assembly and map-based gene cloning^15^,^18^,^19^,^20^,^21^. To our knowledge, 13 QTLs for seed coat color trait, 41 QTLs for plant height, and 17 QTLs for yield related traits have been identified in sesame to date through genetic mapping^15^,^17^,^18^, but there are no reports on the application of linkage mapping techniques to sesame gene cloning.

Of note, since 2010, NGS technology is increasingly being applied to sesame genome assembly^19^,^20^,^21^,^22^,^23^. Construction of an ultra-dense linkage map by whole genome re-sequencing is a key prerequisite for improving advanced genetic and genomic research in sesame^13^,^14^. Here, with aid of improved reference genome data for Yuzhi 11 (PRJNA315784), we constructed an ultra-dense SNP genetic map for sesame using an F_2_ population of a cross between Yuzhi DS899 (*dt1*) and JS012 (*Dt*) and a whole genome re-sequencing approach. *SiDt* and alleles controlling the determinacy of the inflorescence meristem in the three genotypes were identified and validated based on the ultra-dense SNP map and genome re-sequencing data. The findings are valuable for genome assembly, genome-wide association analyses of key agronomic traits, target gene mapping and further studies of *Dt* genes in sesame.

## Results

### Genome re-sequencing and SNP identification

To construct an ultra-dense SNP genetic linkage map for sesame, two parents, cv. Yuzhi DS899 (*dt1*, P_1_) and JS012 (*Dt*, P_2_), and 120 F_2_ progeny were sequenced using an Illumina sequencing approach (sequence data deposited in NCBI, PRJNA 315474) (Table 1) (see Supplementary Table S1). In total, 4.35 billion paired-end raw reads with a length of 100 bp were obtained (Table 1), of which 3.47 gigabase (Gb) of the raw data for Yuzhi D899 had a sequence depth of 18.03×, and 6.38 Gb of the JS012 raw data had a sequence depth of 9.79×. We obtained 4.25 billion reads for the 120 F_2_ progeny, with an average 10.00× depth per sample (Table 1). After applying all the filters, we aligned all high-quality reads from the 122 samples to the reference genome assembly of cv. Yuzhi 11 (240.7 megabase (Mb), PRJNA315784). Results showed that a total of 192,744 SNPs/InDels were detected between Yuzhi DS899 and Yuzhi 11, and 781,528 SNPs/InDels were detected between JS012 and Yuzhi 11. The number of SNPs/ InDels for the F_2_ progeny ranged from 224,327- 624,076, with an average number of 450,101 in each F_2_ progeny genome. All the variants distributed to 748,063 loci and SNP density was high, one SNP/InDel per ~500 bp. Comparative alignments indicated that 706,564 SNPs/InDels (including 623,260 SNPs and 83,304 InDels) in the two parents, representing 94.45% of all detected variants, were high-quality.

### Construction of an ultra-dense SNP linkage map

**Figure 1:**
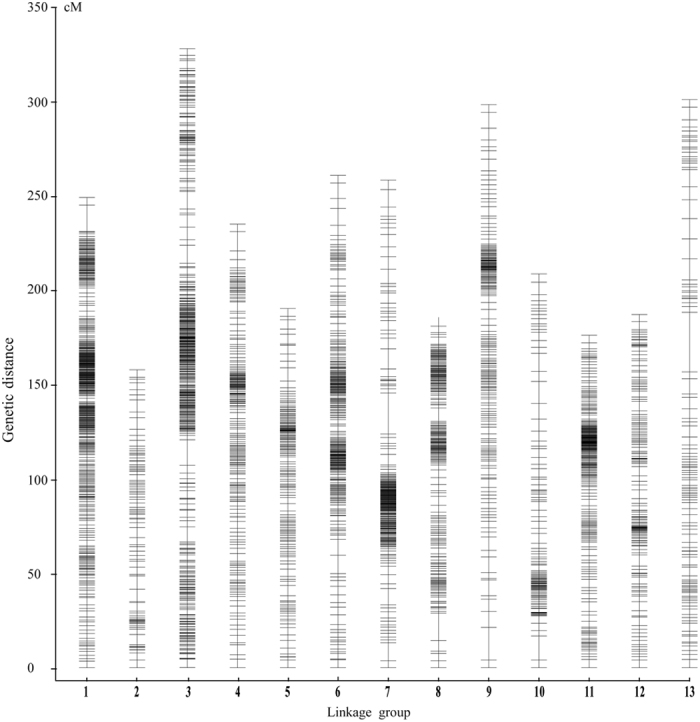
Distribution of SNP markers in 13 linkage groups of the ultra-dense SNP map. Bars indicate SNP markers. The x- axis indicates the linkage group (LG) and the y-axis indicates genetic distance in centi Morgans (cM). A total of 30,193 SNP markers were mapped to the 13 LGs. The 13 LGs were randomly designated as LG1-LG13.

Before constructing an SNP genetic map, heterozygous SNPs among the 623,260 high-quality SNPs from the two parents were filtered and discarded, and common homozygous SNPs in the parents were deleted. A total of 444,731 homozygous unique SNPs were detected in the two parents, 46,767 of which were obtained from Yuzhi DS899 and 397,964 from JS012 (Table 1). The genotypes of all the 444,731 unique loci from the two parents were assigned for each F_2_ progeny. A matrix containing 40,034 skeleton bins (i.e., groups of co-segregating markers) was created for the 120 F_2_ progeny. On the basis of Mendelian separation ratios (Chi-square test, P > 0.05), 3,101 bins containing 30,494 SNP markers were chosen from the above skeleton bins for linkage mapping (see Supplementary Table S2). An ultra-dense SNP genetic map was then constructed by mapping 3,041 of these bin markers containing 30,193 SNP markers (See Supplementary Table S3) to 13 LGs (Fig. 1) (the remaining 60 bin markers were in LGs containing <10 bin markers). The resulting ultra-dense SNP map covered 2,981.28 cM of the sesame genome (Table 2), and the number of SNP markers in each LG ranged from 882 (LG13) to 4,742 (LG3), with a mean of 2,322.62 SNPs per linkage group. The length of the 13 LGs ranged from 152.35 cM (LG2) to 325.49 cM (LG3), with an average size of 229.33 cM, and only 78 gaps larger than 5 cM in length were detected in the map. The average marker density of the whole map is approximately one bin per 0.98 cM or one SNP per 0.10 cM, the groups with the highest and lowest average marker density being LG1 (0.05 cM) and LG13 (0.34 cM), respectively.

### Characteristics and inheritance of the determinate growth habit trait in sesame

**Figure 2:**
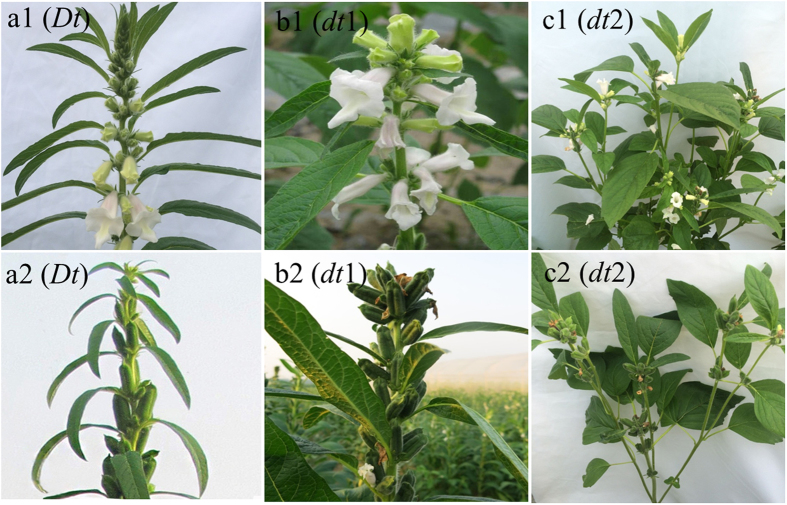
Three inflorescence meristem development phenotypes in sesame. The top and bottom photographs show the stem apexes of three genotypes at flowering and maturity, respectively. (**a1,a2**) Show the indeterminate genotype *Dt* (the wild type). (**b1**,**b2**) Show Yuzhi DS899, a determinate type 1 genotype (*dt1*) with a terminal flower (or capsule) cluster at the shoot apex. (**c1**,**c2**) Show 08TP092, a determinate type 2 genotype (*dt2*) with a terminal flower (or capsule) cluster at apex of each shoot. In *dt2* only 2–3 capsule nodes are formed on both the main stem and each lateral branch.

To date, three inflorescence meristem termination phenotypes have been documented in sesame (Fig. 2) (see Supplementary Table S4). The shoot apex in Yuzhi 11 (*Dt* type) grows indeterminately throughout the whole reproductive period, more than 20 capsule nodes are formed, and plant height reaches 165.0 cm (Fig. 2-a1). In *dt1* and *dt2* types, as development of the apical meristem terminates, a flower cluster develops at the shoot apex and grows into a capsule cluster (Fig. 2-b1). Yuzhi DS899 (*dt1* type) develops 8–20 capsule nodes, and the number of capsule nodes varies with latitude. 08TP092 (*dt2* type) forms only 2–3 capsule nodes on the main stem and each lateral branch, and capsule node number is independent of latitude.

In order to systematically investigate the inheritance of *dt1* and *dt2* genotypes in sesame, we constructed F_1_ hybrids and F_2_ populations of five cross combinations, namely ‘Yuzhi DS899× Yuzhi 11’, ‘08TP092× Yuzhi 11’, ‘Yuzhi DS899× 08TP092’, ‘Yuzhi DS899× Ningbohei’ and a mapping population of ‘Yuzhi DS899 × JS012’ (Table 3). The phenotypes of all eight F_1_ generations from direct crosses and reciprocal crosses between *Dt* and *dt* types were found to be indeterminate, showing that the indeterminate trait (*Dt*) is dominant. In *dt1* backcrosses of ‘Yuzhi DS899× Yuzhi 11’ and ‘Yuzhi DS899× Ningbohei’, the segregation ratios of the indeterminate and determinate (*Dt*:*dt1*) phenotypes fitted the expected 1:1 ratio (Table 3). In addition, the segregation ratio of inflorescence meristem termination in the F_2_ population fitted the expected ratio of 3 (*Dt*):1 (*dt*). Chi-square tests (P > 0.05) validated that the segregation of the determinate growth trait in *dt1* and *dt2* lines fitted the Mendelian inheritance mode, as the x^2^ values for trait segregation in the F_2_ population ranged from 0.22 to 0.53. We also investigated the genetic relationship between *dt1* and *dt2* phenotypes. All progeny of direct and reciprocal crosses, back-crosses and the F_2_ population developed from the ‘Yuzhi DS899 × 08TP092’ cross were found to be determinate (Table 3). Results confirmed that *dt2* (08TP092) is allelic with *dt1* (Yuzhi DS899), and that the two phenotypes are controlled by a regressive gene locus. We annotated the determinacy alleles in Yuzhi DS899 and 08TP092 as *Sidt1* and *Sidt1-2*, respectively.

### *SiDt* locus mapping, gene detection and annotation

**Figure 3:**
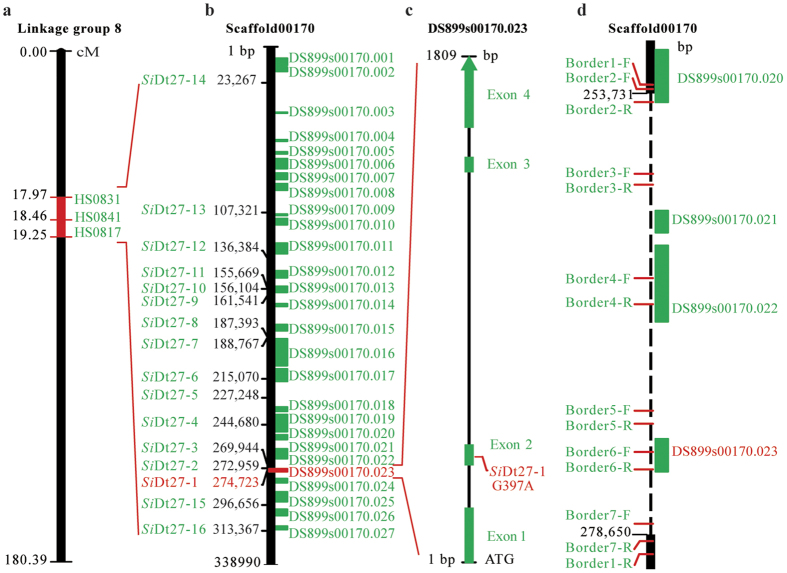
Map-based cloning of *SiDt* and its alleles in sesame. (**a**) Location of the QD1 interval in the SNP map. The QD1 interval is located at 18.2 cM–19.0 cM in LG8 of the ultra-dense SNP map. Three bin markers, HS0831 (17.97 cM), HS0841 (18.46 cM) and HS0817 (19.25 cM), are located in the QD1 window. (**b)** Sequence structure of the QDt1 window in Yuzhi DS899 (*dt1* type). The QDt1 locus in the genome of Yuzhi DS899 corresponds to a physical distance of 292.35 kb (from 2,464 bp–294,813 bp) in scaffold 00170 in which 25 putative genes and 14 unique SNPs/ InDels were detected. Green rectangles refer to putative genes. (**c)** Structure of the DS899s00170.023 gene, the target gene controlling the determinate trait. The DS899s00170.023 gene (in red) is 1,809 bp and comprised of 4 exons (in green) and 3 introns. The G379A SNP site is located in the 2^nd^ exon. (**d)** PCR primer design for cloning the *SiDt* allele in 08TP092 (*dt2* type). According to 08TP092 genomic data, a 24.9 kb fragment from 253,731 bp–278,650 bp (black broken line) in scaffold 00170 has been deleted. Three genes DS899s00170.023, DS899s00170.022, and DS899s00170.021, and part of the sequence of DS899s00170.020 (in green) are covered. Seven primer pairs based on the predicted deleted fragment were designed to verify the deletion of the *SiDt* gene and its flanking sequences.

Using the above ultra-dense SNP map and the phenotypic segregation of the F_2_ progeny from the ‘Yuzhi DS899 × JS012’ cross, we performed gene locus mapping for the determinate growth trait using winQTLcart and QTLNetwork, respectively (See Supplementary Table S5). The logarithm (base 10) of odds (LOD) score for the determinacy trait was set to 4.9 with 1,000 permutations. WinQTLcart analysis results showed that a locus linked to the determinate growth habit was located in the 16.7 cM–22.2 cM inheritance interval of LG8 and had an R^2^ value of 70.2%, while QTLNetwork identified a locus in the 18.0 cM–19.2 cM inheritance interval of LG8 that had a P-value of less than 1E-6 (See Supplementary Fig. S1). Consolidation of the above results indicated that the QDt1 locus for the determinate habit trait in sesame was mapped in the 18.0 cM–19.2 cM inheritance interval in LG 8 (Fig. 3a), which contained 36 SNP loci belonging to 3 bin markers (HS0831, HS0841 and HS 0817) (See Supplementary Table S2 and Table S3). Genome comparison of the QDt1 window was initially performed between Yuzhi DS899 and JS012 to identify the candidate SNP locus derived from Yuzhi DS899 (Fig. 3b). Results showed that the corresponding genome sequences underlying the QDt1 interval were located in Scaffold 00170 (338,990 bp in length) of the Yuzhi 11 reference genome. The physical distance of the QDt1 interval was 292.35 kb and was comprised of 25 predicted protein encoding genes (Fig. 3b; Supplementary Table S6). Genes DS899s00170.026 and DS899s00170.027 were found to be adjacent to the target region. Nine SNPs and five InDels in the 292.35 kb fragment, were uniquely present in the determinacy genotype Yuzhi DS899 (Fig. 3b; Supplementary Table S7). To fine map the target marker, the above unique SNPs and InDels, in addition to 1 SNP and 1 InDel adjacent to the QDt1 interval, were converted into PCR-based SNP/InDel markers and used for association analysis using the two ‘Yuzhi DS899 × JS012’ and ‘Yuzhi DS899× Ningbohei’ F_2_ populations. The coefficients of determination of the above candidate SNPs and InDels ranged from 94.4% to 100.0% in all the 400 progeny of the two F_2_ populations. The SNP marker *SiDt*27-1 located in gene DS899s00170.023 was the only marker that entirely co-segregated with the determinacy trait (See Supplementary Fig. S2).

We subsequently amplified the target gene DS899s00170.023 from Yuzhi 11 and Yuzhi DS899 according to genomic sequence (1, 809 bp in Yuzhi DS899) and *SiDt*27-1 marker information (Fig. 3c; Supplementary Table S8), and named this gene for controlling determinate growth habit as *Sidt1* (See Supplementary Table S6 and Table S9). Non-redundant (NR) protein annotation indicated that the *SiDt* protein is a terminal flower (TFL) like protein. *SiDt* alleles are comprised of 4 exons and 3 introns and code for 531 amino acid residues (Fig. 3c). A SNP, G397A, in the 2^nd^ exon was detected between the *Sidt1* and *SiDt* genes and which caused a change in amino acid from Ser to Asn (S79N) (GenBank accession No. KU240042). Protein homology modelling showed that there is no difference in 3D structure between the *SiDt* and *Sidt*1 proteins (See Supplementary Fig. S3). The PROVEAN score for the S79N change in *SiDt* is −2.706, indicating a deleterious effect on protein functionality.

According to the above genetic analysis, the *dt2* line had the same recessive gene locus for the determinacy trait as the *dt1* mutant line. We attempted to amplify the gene sequence of the *SiDt* allele (named *Sidt1-2* in this study) using the primer pair *SiDt* F and *SiDt* R (see Supplementary Table S2). However, as no amplicon was obtained from 08TP 092, we re-sequenced the genome of 08TP092 (PRJNA316751). A total of 4.76 Gb of raw data were obtained with a sequence depth of 13.46**×**. Genome alignment of Yuzhi 11 and 08TP092 (*dt2*) showed that a ~24.9 kb fragment containing the *SiDt* (DS899s00170.023), DS899s00170.022, and DS899s00170.021 loci and part of the DS899s00170.020 sequence (Fig. 3d) was missing in 08TP092. To further explore the target region, we designed 7 primer pairs based on the nucleotide sequences of the predicted deleted segment and performed PCR amplifications in Yuzhi 11 and 08TP092 (Fig. 3d) (See Supplementary Table S8). With the exception of the 1,008 bp amplicon generated using the border1 primer pair, no amplicons from within the 24.9 kb sequence were obtained in 08TP092 using the remaining 6 primer pairs. These results suggest that the *SiDt* gene has been lost from the genome of line 08TP092 (Fig. 3d).

### *SiDt* homolog detection and gene alignment analysis

We screened for homologous genes of *SiDt* in the genome of Yuzhi 11. Seven *SiDt* gene homologs with a protein identity variation ranging from 29–78% were detected (See Supplementary Table S9 and Fig. S4). Four of these homologs were annotated as TFL-like proteins. Phylogenetic analysis showed that *SiDt* and its 7 homologous proteins can be divided into two groups, group II proteins, Sis00157-2, Sis00154-1 and Sis00141-1 showing high identity to *SiDt. SiDt* and the Sis00154-1 protein in Scaffold00154 of the Yuzhi 11 genome had the highest identity (78%).

To further explore this genetic diversity and to verify the target SNP allele of *SiDt*, we performed gene alignment and DNA polymorphism analysis of *SiDt* sequences in Yuzhi 11, Yuzhi DS899 and 30 indeterminate varieties from a worldwide germplasm bank (See Supplementary Table S10 and Fig. S5). The *SiDt* sequence of 25 the 30 indeterminate varieties (M1-M30) had 100% identity to that of Yuzhi 11, while 18 SNP sites were present in the other 5 varieties, whose *SiDt* sequence identity ranged from 99.06% to 99.28%. All 18 SNP sites in the *SiDt* gene were different from the G397A mutation, and 16 of the 18 sites were located in introns of the *SiDt* gene. We performed DNA polymorphism analysis of the *SiDt* gene using genome re-sequencing data from 715 germplasm resources in public sesame datasets (PRJEB8078 in NCBI), retaining SNP sites detected in ≥7 (1%) samples (See Supplementary Table S11). Sixteen of the 18 SNP sites were found in 35 varieties. All 16 SNPs were present in the 5 parent cultivars (Supplementary Fig. S5). We thus conclude that the G397A SNP in the *SiDt* gene is the sole SNP site between indeterminate and determinate genotypes.

### Alignment of *SiDt* with homologues from other species

**Figure 4:**
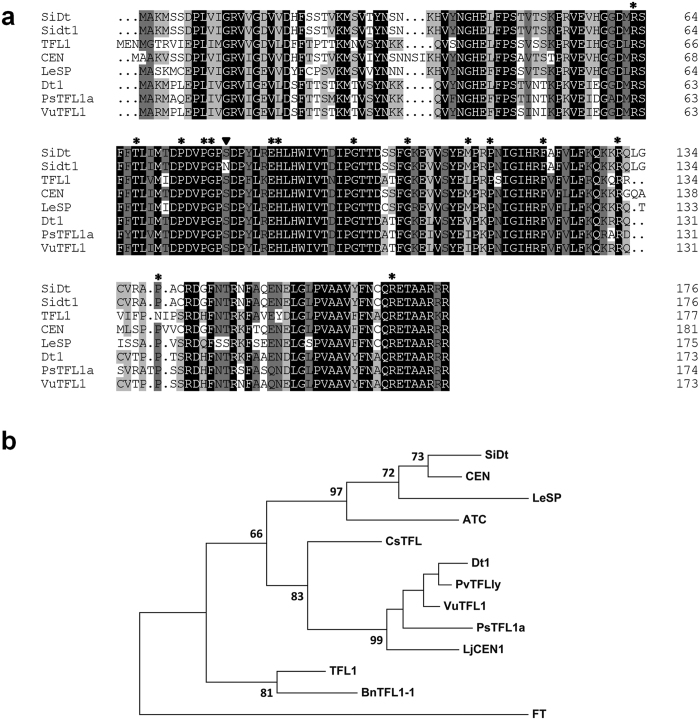
Comparison of the deduced amino acid sequences of *SiDt* and *Sidt1* with their homologues in other plant species. (**a**) Comparison of the predicted amino acid sequences of *SiDt* and *Sidt1 (S. indicum*) with those of TFL1 (*A. thaliana*), CEN (*A. majus*), LeSP (*L. Esculentum*), Dt1 (*G. max*), PsTFL1a (*P. sativum*) and VuTFL1 (*V. unguiculata*). Asterisks above the amino acid sequences indicate amino acid residues which vary among TFL1 homologues (but not in the homologues aligned here). The arrowhead indicates the S79N mutation site in the *Sidt*1 protein. Identical residues are shaded in black; conserved residues are shaded in gray; residues with low identity are shaded in light gray. The black dot indicates an amino acid gap. (**b**) Phylogenetic tree of different TFL-homologs generated using the maximum likelihood method. Numbers above the branches indicate the support with 1000 bootstrap replications. The cluster includes *SiDt* protein and homologs TFL1 (AAB41624, *A. thaliana*), ATC (BAA75932.1, *A. thaliana*), CEN (AAB36112, *A. majus*), LeSP (AAC26161, *L. esculentum*), Dt1 (ADF30893, *Glycine max*), PvTFLly (AFI47668, *P. vulgaris*), PsTFL1a (AAR03725, *P. sativum*), VuTFL1 (AIA10354, *V. unguiculata*), CsTFL (NP_001275848, *C. sinensis*), LjCEN1 (AAQ93599, *L. japonicas*), BnTFL1-1 (BAA33415, *B. napus*) and FT (BAA77838, *A. thaliana*).

Currently, dozens of target genes that control the determinate growth habit, such as the *tfl* gene in *Arabidopsis thaliana*^24^, *PvTFL1y* in common bean (*Phaseolus vulgaris*)^25^ and *Sdt1* in Indian mustard (*Brassica juncea*)^26^, have been reported. To analyse the amino acid structure of *SiDt*, we aligned the predicted sequence of the *SiDt* and *Sidt*1 proteins with six homologues from different plant species (Fig. 4). The only mutation site in *Sidt1*, S79N is not present in other TFL1 homologues and is thus a new mutation site that determines inflorescence meristem development (Fig. 4a). Phylogenetic analysis of 12 homologs of TFL1 showed that *SiDt* has highest homology (88.6%) with CEN in *A. majus* (Fig. 4b).

### Expression profiles of *SiDt* and *Sidt1* in sesame

**Figure 5:**
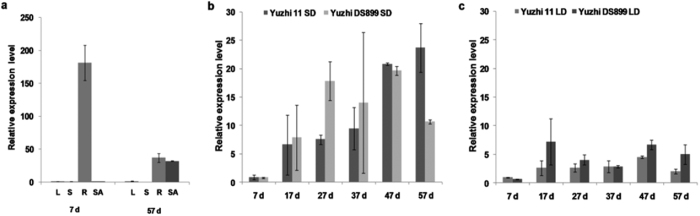
Expression of *SiDt* alleles in indeterminate and determinate lines of sesame. (**a**) Spatial profile of *SiDt* transcription in Yuzhi 11 at the vegetative (7 d) and flowering (57 d) stages under short days (SD) of 12 h light/12 h dark. L, S, R and SA on the x-axis indicate indicate leaf, stem, root and shoot apex tissues, respectively. (**b**) Expression pattern of *SiDt* alleles in the shoot apex tissues of Yuzhi 11 and Yuzhi DS899 from 7–57 d under SD. Plants of 7–37 d were in the vegetative stage. (**c**) Expression pattern of *SiDt* alleles in the shoot apex tissues of Yuzhi 11 and Yuzhi DS899 from 7–57 d under long days (LD) of 15 h light/9 h dark. Plants of 7–57 d were all in the vegetative stage. The *β-tubulin* gene (*SiTUB*, JP631640) was used as an endogenous reference gene for qPCR. *SiDt* and *Sidt1* transcript amount was normalized to *β-tubulin* and analyzed using the ΔΔCt method. Values were calculated from three independent biological replications. The error bar indicates the standard error of the mean.

Sesame is a short-day crop. As photoperiod changes from 12 h to 15 h per day, the budding period in cultivar Yuzhi 11 extends from 40 days after planting to more than 70 d, while the budding period in the *dt1* and *dt2* genotypes extends to 63 d and 58 d, respectively (See Supplementary Table S4). To observe expression of the determinacy gene in sesame, we monitored variation in transcription levels of *SiDt* alleles from 7 d–57 d under various photoperiodic treatments (Fig. 5) using quantitative real-time PCR. In Yuzhi 11, *SiDt* was expressed mainly in root tissues rather than in leaf, stem and shoot apex tissues at early vegetative stage under short days (SD, 12 h light/12 h dark) (Fig. 5a). During the reproductive stage (57 d), the transcription of *SiDt* in the root decreased sharply, while transcription in the shoot apex increased and was close to the transcription level in the root (Fig. 5a). In shoot apex tissues, the expression of *SiDt* in Yuzhi 11 increased from 17 d until 57 d (Fig. 5b). In the *dt1* line, patterns of *Sidt1* transcription were similar to those of *SiDt*, and *Sidt* transcription peaked earlier than *SiDt* transcription in Yuzhi 11. This earlier reduction in *Sidt* transcription may lead to the earlier flowering and termination of inflorescence meristems in the *dt1* mutant (Fig. 5b). Under long days (LD, 15 h light/9 h dark), plantlets of Yuzhi 11 and Yuzhi DS899 (*dt1*) remained in the vegetative stage until 57 d (See Supplementary Table S4). Expression of both *SiDt* alleles in Yuzhi 11 and *Sidt1* in Yuzhi DS899 was weak from 7 d–57 d (Fig. 5c). These results show that *SiDt* alleles are expressed in shoot apical tissues of the wild type and *dt1* mutant mainly during the reproductive stage. In addition, we determined the transcriptional level of the *Dt* gene in the *dt2* genotype. As expected, no transcription of the *Dt* gene in 08TP092 was detected because of the deletion of the *SiDt* gene allele.

## Discussion

In this study, we constructed an ultra-dense SNP genetic linkage map for sesame using whole genome re-sequencing. The 13 LGs matched the 13 chromosomes in the sesame karyotype and can be used for fine genome assembly^21^. Based on our ultra-dense SNP map and genome re-sequencing data, we have shown that the *SiDt* and *Sidt1* alleles control the transition to inflorescence meristem development in the *Dt* and *dt1* sesame genotypes, respectively, and that the other allele of *SiDt* in the *dt2* genotype is missing from the genome. This study is the first example of the application of an ultra-dense SNP genetic map in sesame for QTL mapping and map-based cloning of functional genes involved in key agronomic traits.

Similar to many self-pollinated crops, sesame has a relatively narrow genetic base, as all sesame varieties are derived from the only cultivated sesame species, *S. indicum* L.^17^,^27^,^28^,^29^. There are therefore relatively few universal molecular markers with polymorphisms, such as SSRs and SNPs, in sesame^14^,^15^,^16^,^17^. Three SNP maps for sesame have previously been constructed based on RRGS using the SALF-seq and RAD-seq techniques^16^,^17^,^18^. Only a few thousand SNP markers are currently available and SNP genetic maps of sesame are far from marker saturation. Considering the LD decay distance (150 kb) and genome size (369 Mb by flow cytometry)^15^, an ideal saturated genetic map for sesame would contain more than 2,500 SNPs which are evenly distributed on each of the 13 LGs. We employed a genome re-sequencing strategy using an F_2_ population to construct an ultra-dense SNP genetic map for sesame. The average marker density in our map is approximately one bin per 0.98 cM or one SNP per 0.1 cM (Fig. 1; Table 2). This ultra-dense SNP genetic linkage map can be referred to as a saturated SNP map, in view of the high reliability and high density of the bin markers.

When constructing a saturated genetic map, bin markers representing abundant co-segregating SNPs are always used for genotyping^30^,^31^. Whole genome re-sequencing using a reference genome has previously been applied for the proper alignment and screening of genome sequence tags^9^,^13^,^32^. In this study, we adopted several stringent strategies to improve the accuracy of SNP map construction. Before constructing the data matrix and performing JoinMap analysis, only loci that were called in either parent and ≥20 progeny of the 120 F_2_ population were chosen as high-quality loci. Of these high-quality loci, loci homozygous in both parents were further filtered. In addition, a read depth threshold of ≥5 in each F_2_ progeny was applied for genotype coding.

Here, all 3,101 called bins, containing a total of 30,494 SNP markers, were used for genetic linkage map construction with the minimum LOD score set at 8.0 and the maximum recombination frequency set at 35%. Of the 3,101 bins, 3,041 were mapped onto the linkage map and 60, which were grouped into small LGs with a bin number ranging from 1 to 5, were discarded. Further analysis found that most of the 60 bins were buried in very short contigs, and some of the orphan bins present in longer contigs may have been located near centromere zones and have low recombination rates (data not shown).

During construction of the SNP map, we found that 78 gaps larger than 5 cM were present in the SNP map (Table 2), some of which were located in the middle or close to the middle of LGs and may be centromere regions. Of the 78 gaps, 40 (including 14 gaps >10 cM) were located exactly in junction zones of adjacent contigs. These gaps may be related to the quality of the reference genome assembly, as SNP markers were identified based on these reference sequences, and may be located in highly repetitive sequences or centromeres. The remaining 38 gaps were distributed in contig sequences, and gap size varied from 40 kb to 1.2 Mb and may be due to low recombination frequency in these regions. The exact reason for low recombination in these regions requires further study.

To this point, it has not been possible to perform map-based cloning of sesame genes due to the low marker density of unsaturated genetic maps and limited population size. To determine target SNP sites and clone candidate genes at target intervals, strategies such as increasing the quantity of markers and genome coverage, enlarging population size and using a Mutmap-like strategy are necessary^13^. Here, we located the QDt1 interval associated with the determinate habit trait to LG 8, with an inheritance interval of 18.0 cM–19.2 cM (Fig. 3a) (See Supplementary Table S5). In order to narrow the interval distance, we performed association mapping of 16 SNP/InDel markers in the flanking and adjacent interval using a Mutmap-like strategy (See Supplementary Table S7). As it is tightly linked to the determinate growth habit in sesame, the target *Sidt27-1* SNP marker supplied the necessary information for *SiDt* gene cloning.

Determination of inflorescence meristems is an important developmental transition in crop plants. Here, we systematically investigated the genetic basis of two determinate lines (*dt1* and *dt2*) of sesame. Similar to other determinate mutants or crops^26^,^33^, the inflorescence meristems of the Yuzhi DS899 (*dt1* type) and 08TP092 (*dt2* type) lines were observed to form a terminal flower cluster at the apex. Moreover, the determinate growth habit in the *dt2* type is independent of latitude change (See Supplementary Table S4). Classical genetic analysis showed that the *dt1* and *dt2* genotypes are controlled by a recessive gene locus (Table 3). The first determinate mutant in *Arabidopsis (tfl1-1*) was investigated by Shannon and Meeks-Wagner (1991)^34^. Subsequently, reports showed that *TFL1 (TERMINAL FLOWER 1*) and its orthologs cause early flowering and control inflorescence architecture in *Arabidopsis*^33^,^34^, soybean^35^, Gentian^36^, common bean^25^ and oilseed Brassicas^26^. Another family of FT (*FLOWERING LOCUS T*) genes with high identity to TFL1 was found to control downstream photoperiod-dependent and photoperiod-independent pathways to promote the transition to flowering^27^,^28^,^29^,^30^,^31^,^33^,^34^,^35^,^36^,^37^,^38^,^39^,^40^. Here, we have shown that the DS899s00170.023 gene, named here as *SiDt*, controls the determinate trait in sesame. NR annotation indicated that *SiDt* gene is a TFL1 homolog. Previous reports have indicated that 15 amino acid residues in TFL homologs, such as His88Tyr in TFL1, are important residues for maintaining switch functionality^40^. The G397A mutation identified here in sesame is a new mutation site in TFL homologs and is associated with the conversion of inflorescence meristems between the indeterminate and determinate states (See Supplementary Fig. S5). According to protein structure and PROVEAN analysis, the S79N mutation has no effect on the protein structure of *SiDt*, but protein functionality is likely affected with this deleterious change.

The *SiDt* gene is conserved in sesame germplasm resources. Even though 18 SNP sites were detected in the *SiDt* nucleotide sequences of 30 indeterminate cultivars from the worldwide germplasm collection, only 5 (16.67%) varieties had SNP variants in the *SiDt* gene. Screening of the sesame genome uncovered 7 genes with homology to *SiDt*, protein identity ranging from 29–78%. Three proteins, Sis00157-2, Sis00154-1 and Sis00141-1, whose identity ranged from 52–78%, grouped together with *SiDt* protein, (See Supplementary Fig. S4 and Table S9). To explore the molecular mechanism underlying the transition of vegetative to inflorescence meristems in sesame, these TFL1-like homologs should be studied further.

We used a sequence amplification and genome re-sequencing strategy to identify the *SiDt* allele in the *dt2* line, 08TP092. However, the allelic gene could not be amplified from the nuclear genome (Fig. 3d). In addition to the *Sidt1-2* locus (DS899s00170.023), genes DS899s00170.022, DS899s00170.021 and part of the sequence of DS899s00170.020 were not detected due to the deletion of a 24.9 kb segment. The DS899s00170.022 gene (predicted to be a poly ADP-ribose polymerase 3 protein) is 4,298 bp in length and the DS899s00170.021 gene (predicted to be a CMP-N-acetylneuraminate-beta-galactosamide-alpha-2, 3-sialyltransferase 1-like ADP-ribose polymerase 3) is 1,177 bp, while DS899s00170.020, whose function is unknown, is 2,888 bp in length.

TFL and TFL-like homologues are believed to be negative regulators of flowering time^41^,^42^. According to NR annotation, the *SiDt*/*Sidt*1 has highest identity with the centroradialis protein (CEN) of *Antirrhinum* (Fig. 4). In *Antirrhinum*, CEN, the TFL1 ortholog, is a member of the family of phosphatidylethanolamine-binding proteins (PEBP), is expressed at a late inflorescence phase, and controls the morphological switch between shoot growth and inflorescence development^43^,^44^. In *Arabidopsis*, the expression of TFL1 is weak during early development to delay the commitment to flowering, while expression at later stages increases to maintain the inflorescence meristem^24^. However, in other plant species, the expression levels of TFL1 homologs fluctuate in response to various environmental cues. For example, *GtTFL1* in *Gentiana* is mostly expressed in stems with SAMs (shoot apical meristems) at the vegetative stage. As the plant develops into the reproductive stage, its expression level significantly decreases. Overexpression of *GtTFL1* prevents and delays floral initiation and development^36^. In *Dt1* soybean, the *GmTFL1b* gene is expressed mainly in the root and shoot apical meristems at the vegetative stage. During early vegetative growth, the *GmTLFL1b* transcript accumulates in the SAM and is abruptly lost in the determinate line^35^. In sesame, the *SiDt* transcript is similarly detected mainly in the root and stem apical meristems at the early vegetative and reproductive stages (Fig. 5a,c)^35^. However, the amount of *SiDt* transcript in shoot apex tissues was low in the vegetative stage, both in the wild type and the *dt1* mutant. As plants developed into the reproductive stage, the amount of *SiDt* and *Sidt1* transcript increased to a high level. This increase in *SiDt*/*Sidt1* transcription at the budding phase is similar to the increases in transcription seen in both *TFL1* in *Arabidopsis*^24^ and *CEN* in *Antirrhinum*^43^. The expression pattern of the *SiDt* gene in determinate lines reflects the transition from vegetative to inflorescence meristems. During early development (before 17 d), weak expression delays commitment to flowering, whereas increased expression at later stages (after 27 d) corresponds with the transition to inflorescence meristem development. In addition, we found that, under short days, the transcript peak of *Sidt1* in *dt1* occurred earlier than in *SiDt* (Fig. 5b). This expression pattern is in accordance with the earlier transition to the budding stage in *dt1*. We have shown that the *SiDt* gene cloned here using genetic mapping is the target gene for the inflorescence meristem transition in sesame and plays an important role in the inflorescence meristem termination pathway^25^,^35^.

## Experimental Procedures

### Plant materials and mapping populations

Five germplasm accessions covering the three inflorescence meristem termination phenotypes, i.e., Yuzhi 11, Ningbohei and JS102 (indeterminate, *Dt* type), Yuzhi DS899 (determinate, *dt1*) and 08TP092 (determinate, *dt2*) were chosen to investigate the inheritance of the determinate habit trait in sesame (Table 3, Fig. 2). Yuzhi DS899 was induced from the 91–0 line, a sibline of Chinese sesame cultivar Yuzhi 11 using EMS mutagenesis at the Henan Sesame Research Center, Henan Academy of Agricultural Sciences (HSRC, HAAS) in 2009. The determinate germplasm 08TP092 (*dt2*) was a kind gift from Prof. Amram Ashri of Hebrew University of Jerusalem, Israel in 1984. All the above varieties are available from HSRC, HAAS (see Supplementary Table S4).

The determinate growth habit was investigated at the Sanya (109°50′ E and 18°25′ N), Pingyu (113°62′ E and 32°97′ N) and Yuanyang (113°97′ E and 35°05′ N) experiment stations in 2013–2014. The parents, direct crosses, reciprocal crosses, back-crosses and F_2_ populations derived from the five cross combinations (see Table 3) to investigate phenotypic segregation. The determinate habit trait in each sample was assessed at three or more time points during the flowering and maturation stages. Chi-square tests (P = 0.05) were used to determine the segregation significance for the determinate growth habit. The determinate phenotype was assessed according to the trait description standard described by IPGRI and NBPGR (2004)^45^.

Yuzhi DS899 (*dt1*, P_1_) and JS012 (*Dt*, P_2_) were chosen to construct a mapping F_2_ population (See Supplementary Table S4). A total of 302 F_2_ progeny derived from the ‘Yuzhi DS899 × JS012’ cross were grown at the Sanya Experimental station in 2013–2014. In addition to the two parents, 120 progeny were randomly selected from the F_2_ population for genome re-sequencing and genetic studies. Young leaf tissues were collected, immersed in liquid nitrogen and frozen at −70 °C before genomic DNA extraction.

In addition to Ningbohei (one of the parents), 29 sesame varieties with an indeterminate growth trait were randomly chosen from the core collection of worldwide sesame germplasm at the HRSC for gene diversity analysis (See Supplementary Table S10). Young leaf tissues were collected as above for genomic DNA extraction and PCR amplification.

### Genomic DNA extraction, library construction and sequencing

Genomic DNA was extracted from young leaf tissues of each sample using DNeasy Plant Mini Kits (QIAGEN, Hilden, Germany) and fragmented by sonication. Standard paired-end (PE) libraries were constructed according to Illumina guidelines. DNA ends were polished and an ‘A’ base was then added to each 3′ end. After ligating DNA adaptors with a single ‘T’ base overhang at the 3′ end to the above products, they were purified on 2% agarose gel. Target products with an insert size of 300 bp were excised and purified using Gel Extraction Kits (QIAGEN, Hilden, Germany). Libraries were subsequently sequenced on an Illumina HiSeq2500 platform (Illumina, San Diego, USA) according to standard sequencing protocols.

### Sequencing data analysis and SNP detection

Raw reads obtained from the Illumina HiSeq2500 platform were filtered using Trimmomatic 0.32^46^. The 240.7 Mb Yuzhi 11 genome (PRJNA315784) (version 2), comprised of Illumina data^19^ (version 1) and 454 pyrosequencing data, was used as the reference genome in this study. Alignment of re-sequencing data to the reference genome was performed using BWA 0.7.12 with the default settings described by Li and Durbin (2009)^47^. To obtain high-quality sequences, multiple protocols, including discarding of multiple mapped reads, marking duplicates, recalibrating base quality and InDel realignment, were performed using the Samtools, picardTools and Genome Analysis Tool Kit (GATK) packages, respectively, according to GATK best practice^48^. Putative SNPs and InDels were analyzed for each sample using both Samtools and HaplotypeCaller (in the GATK package) with default settings. The variant calling format (vcf) files from Samtools and GATK for each parent and F_2_ progeny were merged using VCFTools^49^. SNP markers from each of the 120 F_2_ progeny were filtered according to the following high quality (high-confidence) criterion: SNPs presenting 20–100 times in the 120 F_2_ progeny and two parents were retained.

### Creation of a population matrix and linkage map construction

To collect unique and homozygous SNPs, home-made Perl scripts were used to filter the above high-quality SNPs to retain SNPs that were homozygous in either parent,and to delete SNPs present in both parents. Unique P_1_- and P_2_- homozygous SNPs were then used to create a population matrix. The genotypes of the P_1_- or P_2_- unique SNP loci in the 120 F_2_ progeny were coded using home-made Perl scripts according to the following criteria: (1) for homozygous SNPs from P_1_ called in F_2_ progeny, when read depth was ≥5, the SNP was coded as ‘AA’; otherwise, the SNP was coded as ‘D’; (2) for homozygous SNPs from P_2_ called in F_2_ progeny, when the read depth was ≥5, the SNP was coded as ‘BB’; otherwise, the SNP was coded as ‘C’; (3) If SNPs were heterozygous in the F_2_ progeny, they were coded as ‘H’; and (4) if the SNP was not called in the F_2_ progeny, it was regarded as missing and coded as ‘-’. All coded loci were classified in terms of their positions on contigs, and the ‘C’ and ‘D’ genotypes of each F_2_ progeny were corrected using a hidden Markov model approach from the R package MPR^50^ with some modification (the parameter “correct.FUN = correct-FUNHMM” was selected for the correctGeno function). Co-segregated markers were grouped in bins using a home-made Perl script. Chi-square tests (significance level: P > 0.05) were used to test the segregation distortion of the bins. All called bins were utilized in genetic linkage map construction using JoinMap 4.0 (Kyazma, Wageningen, Netherlands) with a minimum LOD score of 8.0 and a maximum recombination value of 0.35 in linkage groups. The marker order in each linkage map was determined using a regression mapping algorithm with an LOD threshold of 3.0. Any LG containing no more than 10 SNP markers was discarded. Marker distance was calculated using Kosambi’s mapping function. LGs were numbered randomly and designated as LG1-LG13.

### Marker-trait association analysis and SNP/InDel marker screening

Locating the gene locus for the determinate habit trait was performed using QTL Cartographer 2.5^51^ and QTLNetwork 2.0^52^. In QTL Cartographer, the CIM method was used to detect the QTL and to estimate its effect^53^. The ultra-dense genetic map was scanned at 2 cM intervals with a window size of 10 cM. LOD significance thresholds (P < 0.05) were determined by running 1,000 permutation tests. The ratio of phenotypic variance explained by genotype was used to determine the peak position of the QTL. In QTLNetwork, additive and epistatic QTLs were detected with a threshold probability of P < 0.005. Critical F values were calculated by running 1,000 permutation tests, and QTL effects were estimated using a Monte Carlo Markov Chain with a Gibbs sample size of 20,000. The genome scan configuration was set up with the following parameters: testing window size: 10 cM, walk speed: 1 cM and filtration window size: 10 cM.

The physical distance of the QDt1 interval locus was determined in the appropriate scaffold of the Yuzhi 11 reference genome (PRJNA315784) based on the bin marker sequences of HS0831 and HS0817. The genomes of Yuzhi DS899 and JS012 were compared to screen unique SNP/InDel sites in the physical distance of the QDt1 interval using home-made Perl scripts. 16 SNP/InDels located in the QDt1 interval and adjacent regions were transformed into PCR-based SNP/InDel markers (See Supplementary Table S8) using the Primer Premier 5.0 program (http://www.premierbiosoft.com/prierdesign/index.html) according to the method of Wei *et al*.^54^. Non- redundant (NR) protein and Kyoto Encyclopedia of Genes and Genomes (KEGG) annotations for candidate genes in Scaffold 00170 were obtained using BLASTP and BLAST2GO, respectively.

### Cloning and annotation of *SiDt* and allele genes

In order to clone the entire gDNA and cDNA sequences of *SiDt* alleles in Yuzhi 11, and the *dt1* and *dt2* genotypes, the *SiDt* F and *SiDt* R primer pair was designed using Primer Premier 5.0 (see Supplementary Table S8). DNA amplification was performed in a 10 μL reaction mixture containing 1× Buffer, 2.0 mmol/L MgCl_2_, 0.1 mmol/L dNTPs, 1 μmol/L of each primer, 0.5 U Taq polymerase and 100~200 ng template DNA. First-strand cDNA synthesis was performed using RevertAid First Strand cDNA Synthesis Kit (Thermo Scientific, Germany) according to the manufacturer’s instruction. Standard PCR reactions were carried out on a PTC-225 machine (MJ Research, USA) under the following conditions: 94 °C for 1 min; 30 cycles of 30 s at 94 °C, 1 min at 55 °C and 1 min at 72 °C, with a final 5 min extension at 72 °C. All PCR products were individually gel purified for Sanger sequencing with three replications.

To identify the *SiDt* allele sequence in *dt2*, the 08TP092 genome was re-sequenced on the Ion Proton^TM^ Sequencer platform (Life technologies, USA) according to the manufactures’ instructions. Raw reads were filtered and aligned with the reference genome of Yuzhi 11 using BWA 0.7.12^47^. To look for the predicted deleted sequences, seven primer pairs (See Supplementary Table S8) were designed based on the 24.9 kb flanking sequences of *SiDt* in Yuzhi 11. PCR reactions were performed using gDNA from Yuzhi 11 and 08TP092 as described above. The cDNA sequence of *SiDt* was sequenced and deposited in the NCBI (GenBank accession No. KU240042).

### *SiDt* homolog detection and analysis of DNA polymorphisms

To identify any *SiDt* homologues in sesame, we scanned the genome of Yuzhi 11 using BLASTP, with a cutoff E value of ≤1E-20 and coverage of ≥50%. To explore DNA polymorphisms and to verify the target SNP allele, *SiDt* sequences from the 30 varieties discussed above (M1-M30) (see Supplementary Table S10) were amplified and then aligned using DNAMAN (http://www.lynnon.com/pc/framepc.html). Genome re-sequencing data from the 715 varieties available in public sesame datasets (PRJEB8078 in NCBI) were used for *SiDt* gene diversity analysis using MEGA 5.2 according to the Muscle method^55^, retaining SNP sites detected in ≥7 (1%) samples. The IDs of 35 samples which had SNPs in the *SiDt* gene are listed in Supplementary Table S11. Phylogenetic trees for the *SiDt* homologs and DNA polymorphism analysis were constructed using the maximum likelihood method^55^. Protein structure and homology-modelling comparisons for *SiDt* and *Sidt*1 proteins were performed using SWISS-MODEL^56^. Whether amino acid changes resulted in changes in protein functionality was evaluated using PROVEAN (http://provean.jcvi.org/index.php). The predefined threshold was set at −2.5.

### Comparison of *SiDt* homologs between sesame and other species

Predicted amino acid sequences of *SiDt* and *Sidt1* were aligned with the 6 homologs of TFL1 (*Arabidopsis thaliana*), CEN (*Antirrhinum majus*), LeSP (*Lycopersicon Esculentum*), Dt1 (*Glycine max*), PsTFL1a (*Pisum sativum*) and VuTFL1 (*Vigna unguiculata*) using DNAMAN (http://www.lynnon.com/pc/framepc.html). A phylogenetic tree was constructed based on 12 TFL1 orthologs in *Arabidopsis* and other 9 crops (see Fig. 4 legend) using the methods described above.

### Photoperiodic treatment

Uniform healthy seeds of Yuzhi 11 (*Dt*), Yuzhi DS899 (*dt1*) and 08TP092 (*dt2*) were sterilized with 3% sodium hypochlorite and grown in plastic pots (20 cm × 20 cm) with sterilized vermiculite and peat (V:V = 3:1) in chambers under a day/night temperature of 28 °C/24 °C with 70 ± 1% relative humidity. Photoperiodic conditions of 15 h light/9 h dark (LD) and 12 h light/12 h dark (SD) were applied using cool intensity light of 100 μmol/m^2^/s. Twenty seeds were sown per pot and there were five pots per genotype per treatment. Each treatment was replicated three times. All treatments were watered regularly with 10-fold-diluted liquid MS medium. Three replicate samples of root, leaf, stem and shoot apical tissues of each genotype were collected at 7, 17, 27, 37, 47 and 57 d after planting. All samples were frozen in liquid nitrogen and stored at −70 °C before extraction of RNA.

### RNA extraction and real time PCR assay of *SiDt* alleles

Total RNA was extracted from tissues using RNAiso Plus Reagent (TaKaRa, Dalian, China). Genomic DNA was removed from the RNA products by DNase I treatment. First-strand cDNA synthesis was performed using a RevertAid First Strand cDNA Synthesis Kit (Thermo Scientific, Germany) according to the manufacturer’s instructions. The primer pair *SiDt*-RT F and *SiDt*-RT R for qRT-PCR amplification of *SiDt* alleles was designed using Primer Premier 5.0. The *β-tubulin* gene was used as an endogenous reference gene^57^. Real-time PCR was performed on a Mastercycler^®^ ep realplex (Eppendorf, Germany) according to the procedures of Wei *et al*.^57^. PCR reactions were carried out in a total volume of 20 μL containing 10 μL FastStart Essential DNA Green Master (Roche, Switzerland) (2×), 2.0 μL diluted first-strand cDNA (5×) and each primer at a final concentration of 0.2 μM. Transcript amount of the *SiDt* and *Sidt1* genes was normalized against the *β-tubulin* gene and analyzed using ΔΔCt method^58^.

### Accession codes

All genomic data generated in this study has been deposited at the National Center for Biotechnology Information (NCBI) under the following accession numbers: (1) PRJNA315784 for the genome of Yuzhi 11 (version 2); PRJNA315474 for the genomes of Yuzhi DS899, JS012 and the 120 F_2_ population; and PRJNA316751 for the genome of 08TP092. The cDNA sequence of *Sidt1* gene has been submitted to NCBI under accession number KU240042.

## Additional Information

**How to cite this article**: Zhang, H. *et al*. Ultra-dense SNP genetic map construction and identification of *SiDt* gene controlling the determinate growth habit in *Sesamum indicum* L. *Sci. Rep.* **6**, 31556; doi: 10.1038/srep31556 (2016).

## Supplementary Material

Supplementary Figures

Supplementary Table S1

Supplementary Table S2-1

Supplementary Table S2-2

Supplementary Table S3

Supplementary Table S4

Supplementary Table S5

Supplementary Table S6

Supplementary Table S7

Supplementary Table S8

Supplementary Table S9

Supplementary Table S10

Supplementary Table S11

## Figures and Tables

**Table 1:** Genome sequencing data for the parents and F_2_ population used for construction of the ultra-dense SNP map.

Sample name	Raw read number	Sequencing Depth (×)^a^	Ratio of high-quality reads		SNP/InDel number	Unique SNP number
			Q ≥ 20	Q ≥ 30		
Yuzhi DS899 (*dt1*) (P_1_)	34,667,944	9.79	95.25%	94.99%	192,744	46,767
JS012 (*Dt*) (P_2_)	63,820,640	18.03	91.78%	91.55%	781,528	397,964
120 F_2_ progeny	4,248,706,712	/	96.10%	91.26%	54,012,159	/
Average	25,405,889	10.00	96.05%	91.29%	450,101	/
Total	4,347,195,296	/	/	/	54,986,431	/

^a^The genome sequencing depth was calculated based on a sesame genome size of 354 Mb, as estimated by K-mer^19^.

**Table 2:** Characteristics of the linkage groups in the ultra-dense SNP map.

LG	Bin number	Marker number	Total length (cM)	Average bin interval/ marker interval (cM)	Gap number (>5 cM in length)
LG1	485	4482	245.85	0.51/0.05	1
LG2	94	1082	152.35	1.62/0.14	6
LG3	499	4742	325.49	0.65/0.07	6
LG4	205	2080	230.51	1.12/0.11	3
LG5	162	1637	185.36	1.14/0.11	3
LG6	286	2923	257.08	0.9/0.09	7
LG7	249	2520	253.96	1.02/0.1	14
LG8	222	2319	180.39	0.81/0.08	3
LG9	205	1808	295.41	1.44/0.16	10
LG10	114	1273	203.81	1.79/0.16	9
LG11	257	2839	170.76	0.66/0.06	1
LG12	151	1606	182.38	1.21/0.11	3
LG13	112	882	297.93	2.66/0.34	12
Total	3041	30193	2981.28	0.98/0.1	78

**Table 3:** Inheritance analysis of the *Dt, dt1* and *dt2* genotypes in sesame.

Cross combination	Indeterminate plant no./Determinate plant no.					Expected ratio	
Parent 1	Parent 2	Direct cross(F_1_)	Reciprocal cross (rF_1_)	BC_1_ (x^2^ value)	F_2_ (x^2^ value)	BC_1_	F_2_
Yuzhi DS899 (*dt1*)	JS012 (*Dt*)	52/0	55/0	97/101 (0.05)	231/71 (0.28)^a^	1:1	3:1
Yuzhi DS899 (*dt1*)	Yuzhi 11 (*Dt*)	45/0	47/0	92/94 (0.01)	215/64 (0.53)	1:1	3:1
08TP092 (*dt2*)	Yuzhi 11 (*Dt*)	52/0	38/0	55/49 (0.24)	177/54 (0.24)	1:1	3:1
Yuzhi DS899 (*dt1*)	08TP092 (*dt2*)	0/37	0/44	/	0/218	/	/
Yuzhi DS899 (*dt1*)	Ningbohei (*Dt*)	64/0	58/0	84/78 (0.15)	229/71 (0.22)	1:1	3:1

x^2^_(0.05, 1)_ = 3.84.

^a^To construct the SNP map, 120 F_2_ progeny (with a segregation ratio (Dt:dt1) of 91/29 and x^2^ value of 0.03) were chosen randomly from the above 302 F_2_ progeny of ‘Yuzhi DS899 × JS012’.

## References

[b1] Bedigian D. & Harlan J. R. Evidence for cultivation of sesame in the ancient world. Econ. Bot. 40, 137–154 (1986).

[b2] Wei L., Miao H., Li C. & Zhang H. Establishment of NMR method for rapid and nondestructive detection of oil content in sesame seeds. Jiangsu Agri. 44, 6–8 (2016).

[b3] Melzer S. . Flowering-time genes modulate meristem determinacy and growth form in *Arabidopsis thaliana*. Nat. genet. 40, 1489–1492 (2008). 18997783 10.1038/ng.253

[b4] Hanano S. & Goto K. Arabidopsis TERMINAL FLOWER1 is involved in the regulation of flowering time and inflorescence development through transcriptional repression. The Plant Cell. 23, 3172–3184 (2011). 21890645 10.1105/tpc.111.088641 PMC3203435

[b5] Ashri A. Sesame (*Sesamum indicum* L.) In Genetic resources, chromosome engineering and crops improvement: oilseed crops (ed. Singh R. J.). 231–280 (CRC, 2006).

[b6] Uzun B. & Çağırgan M. İ. Identification of molecular markers linked to determinate growth habit in sesame. Euphytica. 166, 379–384 (2009).

[b7] Uzun B., Engin Y. & Furat S. Genetic advance, heritability and inheritance in determinate growth habit of sesame. Aust. J. Crop Sci. 7, 978–983 (2013).

[b8] Ashri A. Sesame Breeding in Plant Breeding Reviews (ed. Janick J.) 79–228 (Oxford, 1998).

[b9] Verma P. . Construction of a genetic linkage map and identification of QTLs for seed weight and seed size traits in Lentil (Lens culinaris Medik.). PloS ONE, 10, e0139666 (2015). 26436554 10.1371/journal.pone.0139666 PMC4593543

[b10] Sim S. C. . Development of a large SNP genotyping array and generation of high-density genetic maps in tomato. PLoS ONE. 7, e40563 (2012). 22802968 10.1371/journal.pone.0040563 PMC3393668

[b11] Ganal M. W. . A large maize (*Zea mays* L.) SNP genotyping array: development and germplasm genotyping, and genetic mapping to compare with the B73 reference genome. PLoS ONE. 6, e28334 (2011). 22174790 10.1371/journal.pone.0028334 PMC3234264

[b12] Zhao X. . Loci and candidate gene identification for resistance to *Sclerotinia sclerotiorum* in soybean (Glycine max L. Merr.) via association and linkage maps. The Plant J. 82, 245–255 (2015). 25736370 10.1111/tpj.12810

[b13] Wang S. . Sequence-based ultra-dense genetic and physical maps reveal structural variations of allopolyploid cotton genomes. Genome Biol. 16, 108–108 (2015). 26003111 10.1186/s13059-015-0678-1 PMC4469577

[b14] Wei L. B. . A genetic linkage map construction for sesame (*Sesamum indicum* L.). Genes & genomic. 31, 199–208 (2009).

[b15] Zhang H. . Genetic analysis and QTL mapping of seed coat color in sesame (*Sesamum indicum* L.). PloS ONE. 8, e63898, doi: 10.1371/journal.pone.0063898 (2013). 23704951 PMC3660586

[b16] Zhang Y. . Construction of a high-density genetic map for sesame based on large scale marker development by specific length amplified fragment (SLAF) sequencing. BMC plant biol. 13, 141 (2013). 24060091 10.1186/1471-2229-13-141 PMC3852768

[b17] Wu K. . Genetic analysis and molecular characterization of Chinese sesame (*Sesamum indicum* L.) cultivars using Insertion-Deletion (InDel) and Simple Sequence Repeat (SSR) markers. BMC genet. 15, 35 (2014). 24641723 10.1186/1471-2156-15-35 PMC4234512

[b18] Wang L. . Updated sesame genome assembly and fine mapping of plant height and seed coat color QTLs using a new high-density genetic map. BMC genomics. 17, 1 (2016). 26732604 10.1186/s12864-015-2316-4 PMC4702397

[b19] Zhang H. . Genome sequencing of the important oilseed crop *Sesamum indicum* L. Genome Biol. 14, 401 (2013). 23369264 10.1186/gb-2013-14-1-401 PMC3663098

[b20] Wang L. . Genome sequencing of the high oil crop sesame provides insight into oil biosynthesis. Genome Biol. 15, R39 (2014). 24576357 10.1186/gb-2014-15-2-r39 PMC4053841

[b21] Miao H. The Genome of *Sesamum indicum* L. In *Plant and Animal Genome XXII Conference. Plant and Animal Genome* (2016).

[b22] Miao H. The Sesame Genome Project and Sesame Genome Sequencing. In *Plant and Animal Genome XXII Conference. Plant and Animal Genome* (2014).

[b23] Wei X. . Genetic discovery for oil production and quality in sesame. Nat Commun. 6 (2015). 10.1038/ncomms9609 PMC4634326 26477832

[b24] Bradley D., Ratcliffe O., Vincent C., Carpenter R. & Coen E. Inflorescence commitment and architecture in Arabidopsis. Science. 275, 80–83 (1997). 8974397 10.1126/science.275.5296.80

[b25] Repinski S. L., Kwak M. & Gepts P. The common bean growth habit gene PvTFL1y is a functional homolog of Arabidopsis TFL1. Theor. Appl. Genet. 124, 1539–1547 (2012). 22331140 10.1007/s00122-012-1808-8

[b26] Kaur H. & Banga S. S. Discovery and mapping of *Brassica juncea* Sdt1 gene associated with determinate plant growth habit. Theor. Appl. Genet. 128, 235–245 (2015). 25398617 10.1007/s00122-014-2424-6

[b27] Yue W. D. . Genetic Diversity and Population Structure of Germplasm Resources in Sesame (*Sesamum indicum* L.) by SSR Markers. Acta Agronomica Sinica. 38, 2286–2296 (2012).

[b28] Zhang H. . Development and validation of genic-SSR markers by RNA-seq in sesame. BMC genomics. 13, 316 (2012). 22800194 10.1186/1471-2164-13-316 PMC3428654

[b29] Zhang Y. . Genetic diversity assessment of sesame core collection in China by phenotype and molecular markers and extraction of a mini-core collection. BMC genet. 13, 102 (2012). 23153260 10.1186/1471-2156-13-102 PMC3574832

[b30] Huang X., Feng Q. & Qian Q. High-throughput genotyping by whole-genome resequencing. Genome Res. 19, 1068–1076 (2009). 19420380 10.1101/gr.089516.108 PMC2694477

[b31] Avni R. . Ultra-dense genetic map of durum wheat × wild emmer wheat developed using the 90K iSelect SNP genotyping assay. Mol. Breed. 34, 1549–1562 (2014).

[b32] Zou G. . Identification of QTLs for eight agronomically important traits using an ultra-high-density map based on SNPs generated from high-throughput sequencing in sorghum under contrasting photoperiods. J. Exp. Bot, doi: 10.1093/jxb/ers205 (2012). 22859680

[b33] Ohshima S., Murata M., Sakamoto W., Ogura Y. & Motoyoshi F. Cloning and molecular analysis of the *Arabidopsis* gene Terminal Flower 1. Mol. Gen. Genet. 254, 186–194 (1997). 9108281 10.1007/s004380050407

[b34] Shannon S. & Meeks-Wagner D. R. A mutation in the *Arabidopsis* TFL1 gene affects inflorescence meristem development. Plant Cell. 3, 877–892 (1991). 12324621 10.1105/tpc.3.9.877 PMC160057

[b35] Liu B. . The soybean stem growth habit gene Dt1 is an ortholog of Arabidopsis TERMINAL FLOWER1. Plant Physiol. 153, 198–210 (2010). 20219831 10.1104/pp.109.150607 PMC2862436

[b36] Imamura T., Nakatsuka T., Higuchi A., Nishihara M. & Takahashi H. The gentian orthologs of the FT/TFL1 gene family control floral initiation in Gentiana. Plant Cell Physiol. 52, 1031–1041 (2011). 21531759 10.1093/pcp/pcr055

[b37] Uncu A. Ö., Gultekin V., Allmer J., Frary A. & Doganlar S. Genomic Simple Sequence Repeat Markers Reveal Patterns of Genetic Relatedness and Diversity in Sesame. Plant Genome. 8, 1–12 (2015). 10.3835/plantgenome2014.11.0087 33228311

[b38] Kardailsky I. . Activation tagging of the floral inducer FT. Science. 286, 1962–1965 (1999). 10583961 10.1126/science.286.5446.1962

[b39] Kobayashi H., Kato J., Morioka H., Stewart J. D. & Ohtsuka E. Tryptophan H33 plays an important role in pyrimidine (6–4) pyrimidone photoproduct binding by a high-affinity antibody. Protein Eng. 12, 879–884 (1999). 10556249 10.1093/protein/12.10.879

[b40] Hanzawa Y., Money T. & Bradley D. A single amino acid converts a repressor to an activator of flowering. P. Nat. Acad. Sci. USA. 102, 7748–7753 (2005). 10.1073/pnas.0500932102 PMC1140427 15894619

[b41] Simon R., Igeño M. I. & Coupland G. Activation of floral meristem identity genes in Arabidopsis. Nature. 384, 59–62 (1996). 8900276 10.1038/384059a0

[b42] Mimida N. . Functional divergence of the TFL1-like gene family in Arabidopsis revealed by characterization of a novel homologue. Genes to Cells. 6, 327–336 (2001). 11318875 10.1046/j.1365-2443.2001.00425.x

[b43] Bradley D. . Control of inflorescence architecture in *Antirrhinum*. Nature. 379, 791–797 (1996). 8587601 10.1038/379791a0

[b44] Banfield M. J. & Brady R. L. The structure of *Antirrhinum* centroradialis protein (CEN) suggests a role as a kinase regulator. J. Mol. Biol. 297, 1159–1170 (2000). 10764580 10.1006/jmbi.2000.3619

[b45] IPGRI & NBPGR. In Descriptors for sesame (Sesamum spp.) (eds International Plant Genetic Resources Institute, Italy and National Bureau of Plant Genetic Resources, India) (Rome, 2004).

[b46] Bolger A. M., Lohse M. & Usadel B. Trimmomatic: a flexible trimmer for Illumina sequence data. Bioinformatics. btu170 (2014). 10.1093/bioinformatics/btu170 PMC4103590 24695404

[b47] Li H. & Durbin R. Fast and accurate short read alignment with Burrows–Wheeler transform. Bioinformatics. 25, 1754–1760 (2009). 19451168 10.1093/bioinformatics/btp324 PMC2705234

[b48] Auwera G. A. . From FastQ Data to High-Confidence Variant Calls: The Genome Analysis Toolkit Best Practices Pipeline. Curr. Protoc. Bioform. 43, 11.10.1–11.10.33 (2013). 10.1002/0471250953.bi1110s43 PMC4243306 25431634

[b49] Danecek P. . 1000 Genomes Project Analysis Group. The variant call format and VCFtools. Bioinformatics. 27, 2156–2158 (2011). 21653522 10.1093/bioinformatics/btr330 PMC3137218

[b50] Xie W. . Parent-independent genotyping for constructing an ultrahigh-density linkage map based on population sequencing. P. Natl. Acad. Sci. USA. 107, 10578–83, doi: 10.1073/pnas.1005931107 (2010). PMC2890813 20498060

[b51] Wang S., Basten J. & Zeng Z. Windows QTL Cartographer 2.5. Department of Statistics, North Carolina State University, Raleigh, NC. (http://statgen.ncsu.edu/qtlcart/WQTLCart.htm) (2006).

[b52] Yang J. . QTLNetwork: mapping and visualizing genetic architecture of complex traits in experimental populations. Bioinformatics. 24, 721–723 (2008). 18202029 10.1093/bioinformatics/btm494

[b53] Silva L. C., Wang S. & Zeng Z. B. Composite interval mapping and multiple interval mapping: procedures and guidelines for using Windows QTL Cartographer. Methods Mol. Biol. 871, 75–119, doi: 10.1007/978-1-61779-785-9_6 (2012). 22565834

[b54] Wei L. . Development of SNP and InDel markers via de novo transcriptome assembly in *Sesamum indicum* L. Mol. Breed. 34, 2205–2217 (2014).

[b55] Tamura K. . MEGA5: Molecular Evolutionary Genetics Analysis using Maximum Likelihood, Evolutionary Distance, and Maximum Parsimony Methods. Mol. Biol. Evol. 28, 2731–2739 (2011). 21546353 10.1093/molbev/msr121 PMC3203626

[b56] Biasini M. . SWISS-MODEL: modelling protein tertiary and quaternary structure using evolutionary information. Nucleic Acids Res. 42 (W1), W252–W258 (2014). 24782522 10.1093/nar/gku340 PMC4086089

[b57] Wei L. . Identification and testing of reference genes for sesame gene expression analysis by quantitative real-time PCR. Planta. 237, 873–889 (2013). 23229061 10.1007/s00425-012-1805-9 PMC3579469

[b58] Pfaffl M. W. A new mathematical model for relative quantification in real-time RT-PCR. Nucleic Acids Res. 29, e45 (2001). 11328886 10.1093/nar/29.9.e45 PMC55695

