# Supplementary material for: Ultra-dense SNP genetic map construction and identification of SiDt gene controlling the determinate growth habit in Sesamum indicum L
Source: Sci Rep. 2016 Aug 16;6:31556. doi: 10.1038/srep31556 (PMC4985745; doi:10.1038/srep31556)
Supplement: Supplementary Figures [file srep31556-s1.pdf]

Ultra-dense SNP genetic map construction and identification of *SiDt* gene controlling the determinate growth habit in *Sesamum indicum* L.

Haiyang Zhang*, Hongmei Miao, Chun Li, Libin Wei, Yinghui Duan, Qin Ma, Jingjing Kong, Fangfang Xu and Shuxian Chang

Henan Sesame Research Center, Henan Academy of Agricultural Sciences, Zhengzhou，Henan, 450002, People's Republic of China

* Correspondence should be addressed to Haiyang Zhang (zhy@hnagri.org.cn).

Tel.: 86- 371- 65715936

Fax: 86- 371- 65715936

Postal address: Henan Sesame Research Center, Henan Academy of Agricultural Sciences, Zhengzhou, Henan, 450002, China

Supplementary Fig. S1. QDt1 location for determinate growth habit on LG8 of the ultra-dense SNP map.

Supplementary Fig. S2. Amplification and screening of *Si*Dt 27-1 in F_2 population of the cross between Yuzhi DS899 and Ningbohei. M: DL 2,000 marker with the partial DNA bands of 250 bp and 100 bp; Lane 1-20: 92 bp amplicon of *Si*Dt 27-1 in *dt1* genotype individuals of F_2 population. Lane 21, 22, 28, 29, 31, 35 and 37: 97 bp amplicon of *Si*Dt 27-1 in *Dt* genotype individuals; Lane 23-27, 30, 32-34, 36, 38-40: amplicons of 92 bp and 97 bp in heterozygotic genotype (*Dt*/*dt1*).

Supplementary Fig. S3. Protein homology comparison of 3D structure between the SiDt and Sidt1 proteins. The SNP site of S79N is located at the junction region between different domains. Homology modelling shows that the 3D structures of SiDt (left) and Sidt1 (right) are same.

Supplementary Fig. S4. Phylogenetic analysis of *SiDt* homologues in sesame. Seven homologues of *Sis00046-1*, *Sis00076-1*, *Sis00121-1*, *Sis00157-1*, *Sis00157-2*, *Sis00154-1* and *Sis00141-1* are detected in the Yuzhi 11 genome (PRJNA315784) (version 2). Compared with SiDt, the protein identity of the 7 homologues ranges from 29%- 78%. The phylogenetic tree is constructed using MEGA 5.2 program according to the neighbor-joining (NJ) method. The line unit indicates the bootstrap value of 0.05.

Supplementary Fig. S5. Diversity analysis of *SiDt* among the sesame germplasm resources. Thirty core sesame germplasm are applied for the diversity analysis. A total of 18 SNP sites are detected in *SiDt* sequences among 5 accessions, i.e., M10, M11, M18, M19 and M29 as listed in Supplementary Table S10 online. Asterisks above the nucleotide sequences indicate the SNP sites of *SiDt* (*Sidt*) in Yuzhi 11, YuzhiDS899 and the 30 germplasm. The arrowhead indicates the SNP G397A site between Yuzhi 11 and Yuzhi DS899. The SNPs presented in more than 2 accessions are shaded in black. The

SNPs presented in one specific accession are shaded in grey. Bases in frame are exon sequences of *SiDt*.

Supplementary Table S1. Genome sequencing information of two parents and 120 F_2 progeny in this study. Yuzhi DS899 as parent 1 is determinate (*dt1* type), JS012 as parent 2 is indeterminate (the wild type). A total of 120 F_2 progeny are randomly chosen from the F_2 population of the cross between Yuzhi DS899 and JS012.

Supplementary Table S2. Genotyping matrix information of bin and SNP markers in the F_2 progeny. The information of 3,101 bins and 30,494 SNP markers are listed for linkage mapping.

Supplementary Table S3. Bin marker information of the ultra-density SNP genetic linkage map in sesame. 3,041 bin markers containing 30,193 SNP markers are located in the SNP linkage map.

Supplementary Table S4. Characteristics of the three phenotypes of determinacy growth habit in sesame. The determinacy trait of the five accessions are investigated at Sanya (109 °50' E and 18 °25' N), Pingyu (113 °62' E and 32 °97' N) and Yuanyang (113 °97' E and 35 °05' N) experimental stations in 2013. The average height trait data listed in the table are investigated at Yuanyang experimental station in 2013. Photoperiodic treatments of SD (12h light/12h dark) and LD (15h light/9h dark) are performed in growing chamber under the day/night temperature of 28 ℃/24 ℃ with 70 ±1% relative humidity.

Supplementary Table S5. Location of QDt1 associated with the determinate growth habit in sesame using winQTLCart and QTLNetwork.

Supplementary Table S6. Annotation of all the genes in the QDt1 interval of Yuzhi DS899. Twenty five putative genes are detected in the physical distance corresponding to the QDt1 interval using BLASTP program. DS899s00170.026 and DS899s00170.027 listed in the table are adjacent genes to the QDt1 interval. *'F' refers to forward direction and 'R' refers to reverse direction. KEGG refers to Kyoto Encyclopedia of Genes and Genomes.

Supplementary Table S7. Genotypic variation and validation of the candidate SNP/InDel sites in sesame. Nine SNPs and 5 InDels are detected in the QDt1 interval. The *Si*Dt27-15 SNP and the *Si*Dt27-16 InDel listed in the table are detected at the adjacent flanking region of the QDt1 window. A total of 400 progeny of two F_2 populations of 'Yuzhi DS899 × JS012' and 'Yuzhi DS899 × Ningbohei' are used for screening target SNP/InDel marker.

Supplementary Table S8. Primer information of *SiDt* alleles for gene amplification and for real time PCR in sesame. *β-Tubulin* in sesame (*SiTUB*, JP631640) is used as the endogenous reference gene for qPCR.

Supplementary Table S9. Global screening of *SiDt* homologues in the Yuzhi 11 genome. Seven homologues are detected using BLASTP, with the cutoff of E value ≤1E-20 and the coverage of ≥ 50%.

Supplementary Table S10. *SiDt* gene diversity analysis in sesame germplasm resources using PCR amplification. *SiDt* sequences in 30 sesame germplasm resources (M1-M30) are compared using DNAMAN software, in addition to the parents of the mapping population in the study.

Supplementary Table S11. Diversity analysis of *SiDt* gene in sesame germplasm resources using public genome re-sequencing data. The public genome data of 715 accessionss are collected from PRJEB8078 in NCBI dataset.

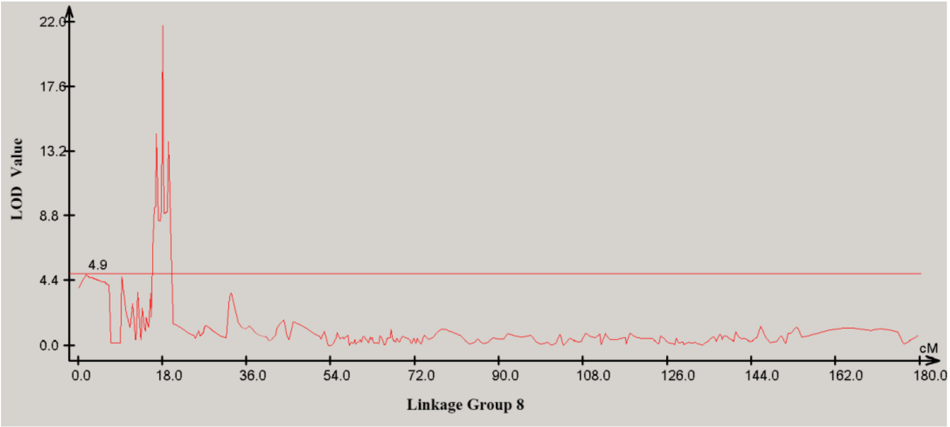

Supplementary Fig. S1. QDt1 location for determinate growth habit on LG8 of the ultra-dense SNP map. LOD refers to the Logistic Organ Dysfunction score. To determine the locus associated with the determinate trait locus, the LOD value is set at 4.9 using 1,000 permutations.

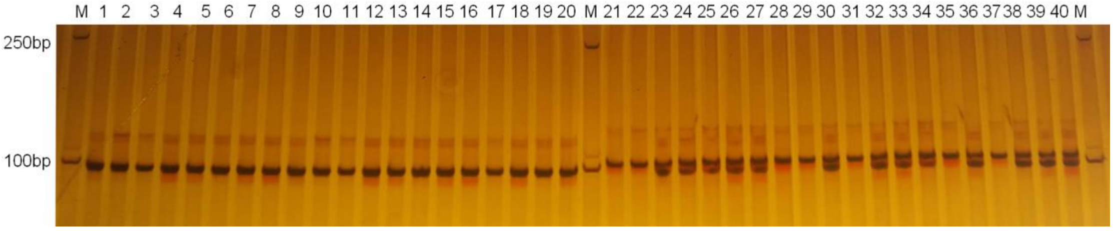

Supplementary Fig. S2. Amplification and screening of *Si*Dt 27-1 in F_2 population of the cross between Yuzhi DS899 and Ningbohei. M: DL 2,000 marker with the partial DNA bands of 250 bp and 100 bp; Lane 1-20: 92 bp amplicon of *Si*Dt 27-1 in *dt1* genotype individuals of F_2 population. Lane 21, 22, 28, 29, 31, 35 and 37: 97 bp amplicon of *Si*Dt 27-1 in *Dt* genotype individuals; Lane 23-27, 30, 32-34, 36, 38-40: amplicons of 92 bp and 97 bp in heterozygotic genotype (*Dt*/*dt1*).

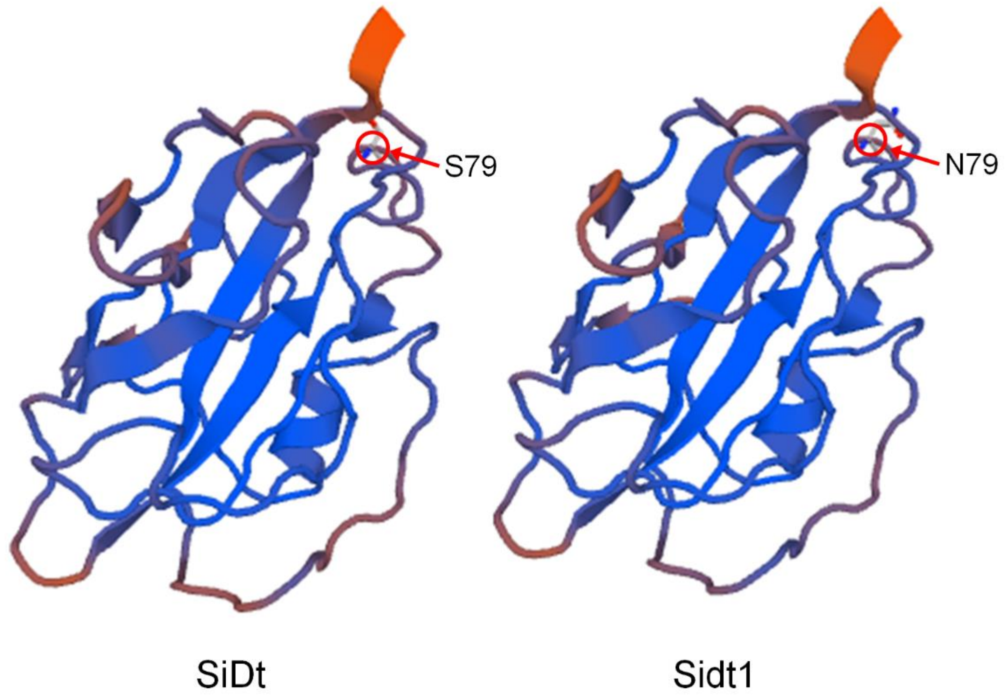

Supplementary Fig. S3. Protein homology comparison of 3D structure between the SiDt and Sidt1 proteins. The SNP site of S79N (in red cycle) is located at the junction region between different domains. Homology modelling shows that the 3D structures of SiDt (left) and Sidt1 (right) are same.

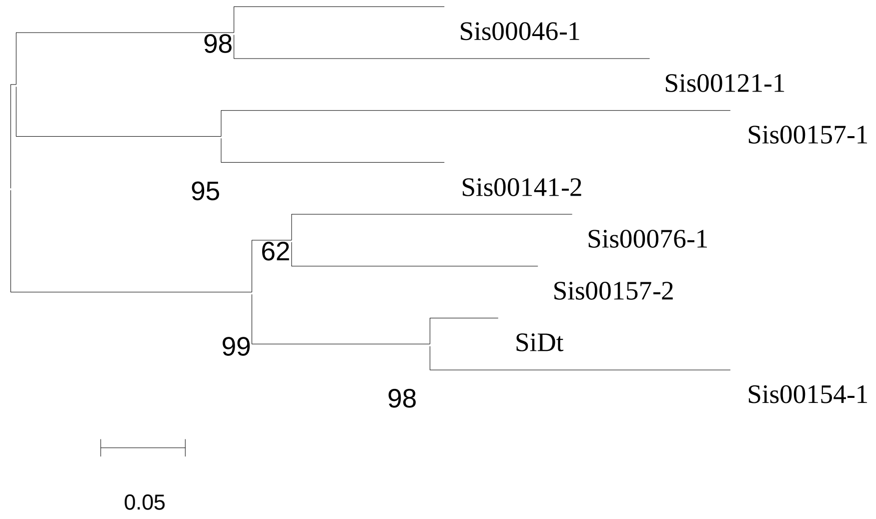

Supplementary Fig. S4. Phylogenetic analysis of *SiDt* homologues in sesame. Seven homologues of *Sis00046-1*, *Sis00076-1*, *Sis00121-1*, *Sis00157-1*, *Sis00157-2*, *Sis00154-1* and *Sis00141-1* are detected in the Yuzhi 11 genome (PRJNA315784) (version 2). Compared with SiDt, the protein identity of the 7 homologues ranges from 29%- 78%. The phylogenetic tree is constructed using MEGA 5.2 program according to the neighbor-joining (NJ) method. The line unit indicates the bootstrap value of 0.05.

Supplementary Fig. S5-1

```
                                                          *
Yuzhi 11        ATGGCAAAAATGTCATCGGACCCCCTTGTGATCGGTAGGGTGGTCGGAGACGTTGTCGATCATTTCTCCTCCACCGTCAAAATGTCAGTCA    91
Yuzhi_Ds899     ATGGCAAAAATGTCATCGGACCCCCTTGTGATCGGTAGGGTGGTCGGAGACGTTGTCGATCATTTCTCCTCCACCGTCAAAATGTCAGTCA    91
M10             ATGGCAAAAATGTCATCGGACCCCCTTGTGATCGGTAGGGTGATCGGAGACGTTGTCGATCATTTCTCCTCCACCGTCAAAATGTCAGTCA    91
M11             ATGGCAAAAATGTCATCGGACCCCCTTGTGATCGGTAGGGTGATCGGAGACGTTGTCGATCATTTCTCCTCCACCGTCAAAATGTCAGTCA    91
M18             ATGGCAAAAATGTCATCGGACCCCCTTGTGATCGGTAGGGTGATCGGAGACGTTGTCGATCATTTCTCCTCCACCGTCAAAATGTCAGTCA    91
M19             ATGGCAAAAATGTCATCGGACCCCCTTGTGATCGGTAGGGTGATCGGAGACGTTGTCGATCATTTCTCCTCCACCGTCAAAATGTCAGTCA    91
M29             ATGGCAAAAATGTCATCGGACCCCCTTGTGATCGGTAGGGTGATCGGAGACGTTGTCGATCATTTCTCCTCCACCGTCAAAATGTCAGTCA    91

Yuzhi 11        CTTACAACTCCAACAAGCATGTCTACAATGGCCATGAGCTCTTCCCTTCCACAGTCACCTCTAAACCTAGGGTTGAGGTCCATGGTGGTGA    182
Yuzhi_Ds899     CTTACAACTCCAACAAGCATGTCTACAATGGCCATGAGCTCTTCCCTTCCACAGTCACCTCTAAACCTAGGGTTGAGGTCCATGGTGGTGA    182
M10             CTTACAACTCCAACAAGCATGTCTACAATGGCCATGAGCTCTTCCCTTCCACAGTCACCTCTAAACCTAGGGTTGAGGTCCATGGTGGTGA    182
M11             CTTACAACTCCAACAAGCATGTCTACAATGGCCATGAGCTCTTCCCTTCCACAGTCACCTCTAAACCTAGGGTTGAGGTCCATGGTGGTGA    182
M18             CTTACAACTCCAACAAGCATGTCTACAATGGCCATGAGCTCTTCCCTTCCACAGTCACCTCTAAACCTAGGGTTGAGGTCCATGGTGGTGA    182
M19             CTTACAACTCCAACAAGCATGTCTACAATGGCCATGAGCTCTTCCCTTCCACAGTCACCTCTAAACCTAGGGTTGAGGTCCATGGTGGTGA    182
M29             CTTACAACTCCAACAAGCATGTCTACAATGGCCATGAGCTCTTCCCTTCCACAGTCACCTCTAAACCTAGGGTTGAGGTCCATGGTGGTGA    182

Yuzhi 11        TATGAGATCATTTTTCACCCTGGTAACAATAATTTTACTAATGATTTTTTACACACCCCACTACTTCTTTCTTCTAATTTCTGCTGATATT    273
Yuzhi_Ds899     TATGAGATCATTTTTCACCCTGGTAACAATAATTTTACTAATGATTTTTTACACACCCCACTACTTCTTTCTTCTAATTTCTGCTGATATT    273
M10             TATGAGATCATTTTTCACCCTGGTAACAATAATTTTACTAATGATTTTTTACACACCCCACTACTTCTTTCTTCTAATTTCTGCTGATATT    273
M11             TATGAGATCATTTTTCACCCTGGTAACAATAATTTTACTAATGATTTTTTACACACCCCACTACTTCTTTCTTCTAATTTCTGCTGATATT    273
M18             TATGAGATCATTTTTCACCCTGGTAACAATAATTTTACTAATGATTTTTTACACACCCCACTACTTCTTTCTTCTAATTTCTGCTGATATT    273
M19             TATGAGATCATTTTTCACCCTGGTAACAATAATTTTACTAATGATTTTTTACACACCCCACTACTTCTTTCTTCTAATTTCTGCTGATATT    273
M29             TATGAGATCATTTTTCACCCTGGTAACAATAATTTTACTAATGATTTTTTACACACCCCACTACTTCTTTCTTCTAATTTCTGCTGATATT    273

Yuzhi 11        TTTTTGGGAGTTTGTGTAAATTAAAGTAGTACGTGCAAATATTTCGTGGGTTTTCAGTAATAATTTCCTCGTTTATATATATGTTTGATCA    364
Yuzhi_Ds899     TTTTTGGGAGTTTGTGTAAATTAAAGTAGTACGTGCAAATATTTCGTGGGTTTTCAGTAATAATTTCCTCGTTTATATATATGTTTGATCA    364
M10             TTTTTGGGAGTTTGTGTAAATTAAAGTAGTACGTGCAAATATTTCGTGGGTTTTCAGTAATAATTTCCTCGTTTATATATATGTTTGATCA    364
M11             TTTTTGGGAGTTTGTGTAAATTAAAGTAGTACGTGCAAATATTTCGTGGGTTTTCAGTAATAATTTCCTCGTTTATATATATGTTTGATCA    364
M18             TTTTTGGGAGTTTGTGTAAATTAAAGTAGTACGTGCAAATATTTCGTGGGTTTTCAGTAATAATTTCCTCGTTTATATATATGTTTGATCA    364
M19             TTTTTGGGAGTTTGTGTAAATTAAAGTAGTACGTGCAAATATTTCGTGGGTTTTCAGTAATAATTTCCTCGTTTATATATATGTTTGATCA    364
M29             TTTTTGGGAGTTTGTGTAAATTAAAGTAGTACGTGCAAATATTTCGTGGGTTTTCAGTAATAATTTCCTCGTTTATATATATGTTTGATCA    364

                                                ▼
Yuzhi 11        GATCATGACAGACCCTGATGTTCCTGGTCCTAGTGATCCATATCTGAGGGAGCACCTGCACTGGTATGCTTTCATTTTTAACTGCTTAAGA    455
Yuzhi_Ds899     GATCATGACAGACCCTGATGTTCCTGGTCCTAATGATCCATATCTGAGGGAGCACCTGCACTGGTATGCTTTCATTTTTAACTGCTTAAGA    455
M10             GATCATGACAGACCCTGATGTTCCTGGTCCTAGTGATCCATATCTGAGGGAGCACCTGCACTGGTATGCTTTCATTTTTAACTGCTTAAGA    455
M11             GATCATGACAGACCCTGATGTTCCTGGTCCTAGTGATCCATATCTGAGGGAGCACCTGCACTGGTATGCTTTCATTTTTAACTGCTTAAGA    455
M18             GATCATGACAGACCCTGATGTTCCTGGTCCTAGTGATCCATATCTGAGGGAGCACCTGCACTGGTATGCTTTCATTTTTAACTGCTTAAGA    455
M19             GATCATGACAGACCCTGATGTTCCTGGTCCTAGTGATCCATATCTGAGGGAGCACCTGCACTGGTATGCTTTCATTTTTAACTGCTTAAGA    455
M29             GATCATGACAGACCCTGATGTTCCTGGTCCTAGTGATCCATATCTGAGGGAGCACCTGCACTGGTATGCTTTCATTTTTAACTGCTTAAGA    455

                                                       *                    *
Yuzhi 11        CCTGATTGATTTAATAAACTAGTATTCTTCAAAAAACTAGAGTTTTGGAATGATCAACACCCTTTTCTCATCCAAGAGGGTAATCAACCAC    546
Yuzhi_Ds899     CCTGATTGATTTAATAAACTAGTATTCTTCAAAAAACTAGAGTTTTGGAATGATCAACACCCTTTTCTCATCCAAGAGGGTAATCAACCAC    546
M10             CCTGATTGATTTAATAAACTAGTATTCTTCAAAAAACTAGAGTTTTGGAATGATCAACACCCTTTTCTCATCCAAGAGGGTAATCAACCAC    546
M11             CCTGATTGATTTAATAAACTAGTATTCTTCAAAAAACTACAGTTTTGGAATGATCAACACCTTTTTCTCATCCAAGAGGGTAATCAACCAC    546
M18             CCTGATTGATTTAATAAACTAGTATTCTTCAAAAAACTACAGTTTTGGAATGATCAACACCTTTTTCTCATCCAAGAGGGTAATCAACCAC    546
M19             CCTGATTGATTTAATAAACTAGTATTCTTCAAAAAACTACAGTTTTGGAATGATCAACACCTTTTTCTCATCCAAGAGGGTAATCAACCAC    546
M29             CCTGATTGATTTAATAAACTAGTATTCTTCAAAAAACTACAGTTTTGGAATGATCAACACCTTTTTCTCATCCAAGAGGGTAATCAACCAC    546

                                                    *
Yuzhi 11        AACACACTTTTTAGGGTTTTTCTTTTAGTTTTTTTAAAAAATTATGGTAGAAACCGTTATTGAAATAGTCTATTGGAGGGGTCGATCATA    637
Yuzhi_Ds899     AACACACTTTTTAGGGTTTTTCTTTTAGTTTTTTTAAAAAATTATGGTAGAAACCGTTATTGAAATAGTCTATTGGAGGGGTCGATCATA    637
M10             AACACACTTTTTAGGGTTTTTCTTTTAGTTTTTTTATAAAATTATGGTAGAAACCGTTATTGAAATAGTCTATTGGAGGGGTCGATCATA    637
M11             AACACACTTTTTAGGGTTTTTCTTTTAGTTTTTTTATAAAATTATGGTAGAAACCGTTATTGAAATAGTCTATTGGAGGGGTCGATCATA    637
M18             AACACACTTTTTAGGGTTTTTCTTTTAGTTTTTTTATAAAATTATGGTAGAAACCGTTATTGAAATAGTCTATTGGAGGGGTCGATCATA    637
M19             AACACACTTTTTAGGGTTTTTCTTTTAGTTTTTTTATAAAATTATGGTAGAAACCGTTATTGAAATAGTCTATTGGAGGGGTCGATCATA    637
M29             AACACACTTTTTAGGGTTTTTCTTTTAGTTTTTTTATAAAATTATGGTAGAAACCGTTATTGAAATAGTCTATTGGAGGGGTCGATCATA    637

Yuzhi 11        TATATATATATATATGTATGTGTTGGGTGGACTATAAAATTGAAGAGAAATTAATTTTAGAAAAAGGAGATTAAAACGTAATTCTAAATGC    728
Yuzhi_Ds899     TATATATATATATATGTATGTGTTGGGTGGACTATAAAATTGAAGAGAAATTAATTTTAGAAAAAGGAGATTAAAACGTAATTCTAAATGC    728
M10             TATATATATATATATGTATGTGTTGGGTGGACTATAAAATTGAAGAGAAATTAATTTTAGAAAAAGGAGATTAAAACGTAATTCTAAATGC    728
M11             TATATATATATATATGTATGTGTTGGGTGGACTATAAAATTGAAGAGAAATTAATTTTAGAAAAAGGAGATTAAAACGTAATTCTAAATGC    728
M18             TATATATATATATATGTATGTGTTGGGTGGACTATAAAATTGAAGAGAAATTAATTTTAGAAAAAGGAGATTAAAACGTAATTCTAAATGC    728
M19             TATATATATATATATGTATGTGTTGGGTGGACTATAAAATTGAAGAGAAATTAATTTTAGAAAAAGGAGATTAAAACGTAATTCTAAATGC    728
M29             TATATATATATATATGTATGTGTTGGGTGGACTATAAAATTGAAGAGAAATTAATTTTAGAAAAAGGAGATTAAAACGTAATTCTAAATGC    728

                                                                                        *
Yuzhi 11        AAACAAATTTAAATACTATTGCATTACAATTTTAAGATTTCTCTGCAAAAAACTAAGCTCCTAGGAAAGTTCGTTCACTTTAAGGTACAAG    819
Yuzhi_Ds899     AAACAAATTTAAATACTATTGCATTACAATTTTAAGATTTCTCTGCAAAAAACTAAGCTCCTAGGAAAGTTCGTTCACTTTAAGGTACAAG    819
M10             AAACAAATTTAAATACTATTGCATTACAATTTTAAGATTTCTCTGCAAAAAACTAAGCTCCTAGGAAAGTTCATTCACTTTAAGGTACAAG    819
M11             AAACAAATTTAAATACTATTGCATTACAATTTTAAGATTTCTCTGCAAAAAACTAAGCTCCTAGGAAAGTTCATTCACTTTAAGGTACAAG    819
M18             AAACAAATTTAAATACTATTGCATTACAATTTTAAGATTTCTCTGCAAAAAACTAAGCTCCTAGGAAAGTTCATTCACTTTAAGGTACAAG    819
M19             AAACAAATTTAAATACTATTGCATTACAATTTTAAGATTTCTCTGCAAAAAACTAAGCTCCTAGGAAAGTTCATTCACTTTAAGGTACAAG    819
M29             AAACAAATTTAAATACTATTGCATTACAATTTTAAGATTTCTCTGCAAAAAACTAAGCTCCTAGGAAAGTTCATTCACTTTAAGGTACAAG    819

Yuzhi 11        ATGTATTATAAAATTTTTTTGGTGATAATACATGAGATTCGTAAGTTTAGAGTGATGGTCTACACTACATGTGATGTAAGGTCCACAAATC    910
Yuzhi_Ds899     ATGTATTATAAAATTTTTTTGGTGATAATACATGAGATTCGTAAGTTTAGAGTGATGGTCTACACTACATGTGATGTAAGGTCCACAAATC    910
M10             ATGTATTATAAAATTTTTTTGGTGATAATACATGAGATTCGTAAGTTTAGAGTGATGGTCTACACTACATGTGATGTAAGGTCCACAAATC    910
M11             ATGTATTATAAAATTTTTTTGGTGATAATACATGAGATTCGTAAGTTTAGAGTGATGGTCTACACTACATGTGATGTAAGGTCCACAAATC    910
M18             ATGTATTATAAAATTTTTTTGGTGATAATACATGAGATTCGTAAGTTTAGAGTGATGGTCTACACTACATGTGATGTAAGGTCCACAAATC    910
M19             ATGTATTATAAAATTTTTTTGGTGATAATACATGAGATTCGTAAGTTTAGAGTGATGGTCTACACTACATGTGATGTAAGGTCCACAAATC    910
M29             ATGTATTATAAAATTTTTTTGGTGATAATACATGAGATTCGTAAGTTTAGAGTGATGGTCTACACTACATGTGATGTAAGGTCCACAAATC    910
```

Supplementary Fig. S5-2

```
Yuzhi 11      CCGCATCATATGTGTCAGACGCACTACGAAAAACCCTTGTTTCATCCTATGAAAAAAGTAGGTTTCATCGAGTCACTTCCAACTAAATCTC   1001
Yuzhi_Ds899   CCGCATCATATGTGTCAGACGCACTACGAAAAACCCTTGTTTCATCCTATGAAAAAAGTAGGTTTCATCGAGTCACTTCCAACTAAATCTC   1001
M10           CCGCATCATATGTGTCAGACGCACTACGAAAAACCCTTGTTTCATCCTATGAGAAAAGTAGGTTTCATCGAGTCACTTCCAACTAAATCTC   1001
M11           CCGCATCATATGTGTCAGACGCACTACGAAAAACCCTTGTTTCATCCTATGAGAAAAGTAGGTTTCATCGAGTCACTTCCAACTAAATCTC   1001
M18           CCGCATCATATGTGTCAGACGCACTACGAAAAACCCTTGTTTCATCCTATGAGAAAAGTAGGTTTCATCGAGTCACTTCCAACTAAATCTC   1001
M19           CCGCATCATATGTGTCAGACGCACTACGAAAAACCCTTGTTTCATCCTATGAGAAAAGTAGGTTTCATCGAGTCACTTCCAACTAAATCTC   1001
M29           CCGCATCATATGTGTCAGACGCACTACGAAAAACCCTTGTTTCATCCTATGAGAAAAGTAGGTTTCATCGAGTCACTTCCAACTAAATCTC   1001

Yuzhi 11      TGTCCAAGTGATGATCCATGAAAATGTTGTTTGTCGTGTCGACTATCCTTCATTTCATTATTCCTATGGAAAATTACCCTTTAAAAATATA   1092
Yuzhi_Ds899   TGTCCAAGTGATGATCCATGAAAATGTTGTTTGTCGTGTCGACTATCCTTCATTTCATTATTCCTATGGAAAATTACCCTTTAAAAATATA   1092
M10           TGTCCAAGTGATGATCCATGAAAATGTTGTTTGTCGTGTCGACTATCCTTCATTTCATTATGCCTATGGAAAATTACCCTTTAAAAATATA   1092
M11           TGTCCAAGTGATGATCCATGAAAATGTTGTTTGTCGTGTCGACTATCCTTCATTTCATTATGCCTATGGAAAATTACCCTTTAAAAATATA   1092
M18           TGTCCAAGTGATGATCCATGAAAATGTTGTTTGTCGTGTCGACTATCCTTCATTTCATTATGCCTATGGAAAATTACCCTTTAAAAATATA   1092
M19           TGTCCAAGTGATGATCCATGAAAATGTTGTTTGTCGTGTCGACTATCCTTCATTTCATTATGCCTATGGAAAATTACCCTTTAAAAATATA   1092
M29           TGTCCAAGTGATGATCCATGAAAATGTTGTTTGTCGTGTCGACTATCCTTCATTTCATTATGCCTATGGAAAATTACCCTTTAAAAATATA   1092

Yuzhi 11      CTCTTGCCTGGTTCCACGTCTTCTATTTCAACATCGTATTATCTGATTGTTGAATAGTGATAATTTGACTGTCGAAATTACATAAAGCACA   1183
Yuzhi_Ds899   CTCTTGCCTGGTTCCACGTCTTCTATTTCAACATCGTATTATCTGATTGTTGAATAGTGATAATTTGACTGTCGAAATTACATAAAGCACA   1183
M10           CTCTTGCCTGGTTCCACATCTTCTATTTCAACATCGTATTATCTGATTGTTGAATAGTGATAATTTGACTGTTGAAATTAGATAAAGCATA   1183
M11           CTCTTGCCTGGTTCCACATCTTCTATTTCAACATCGTATTATCTGATTGTTGAATAGTGATAATTTGACTGTTGAATTTAGATAAAGCATA   1183
M18           CTCTTGCCTGGTTCCACATCTTCTATTTCAACATCGTATTATCTGATTGTTGAATAGTGATAATTTGACTGTTGAAATTAGATAAAGCATA   1183
M19           CTCTTGCCTGGTTCCACATCTTCTATTTCAACATCGTATTATCTGATTGTTGAATAGTGATAATTTGACTGTTGAAATTAGATAAAGCATA   1183
M29           CTCTTGCCTGGTTCCACATCTTCTATTTCAACATCGTATTATCTGATTGTTGAATAGTGATAATTTGACTGTTGAAATTAGATAAAGCATA   1183

Yuzhi 11      ATATTAAAATAGAATAACTAAATCTGTATCGGATTCTCAATTGTTTACCCTAGTTAAAAAAATATTGCTTATATATGTGGCTTGACAAATG   1274
Yuzhi_Ds899   ATATTAAAATAGAATAACTAAATCTGTATCGGATTCTCAATTGTTTACCCTAGTTAAAAAAATATTGCTTATATATGTGGCTTGACAAATG   1274
M10           ATATTAAAATAGAATATCTAAATCTGTATCGGATTCTCAATTGTTTACCCTAGTTAAAAAAATATTGCTTATATATGTGGCTTGACAAATG   1274
M11           ATATTAAAATAGAATATCTAAATCTGTATCGGATTCTCAATTGTTTACCCTAGTTAAAAAAATATTGCTTATATATGTGGCTTGACAAATG   1274
M18           ATATTAAAATAGAATATCTAAATCTGTATCGGATTCTCAATTGTTTACCCTAGTTAAAAAAATATTGCTTATATATGTGGCTTGACAAATG   1274
M19           ATATTAAAATAGAATATCTAAATCTGTATCGGATTCTCAATTGTTTACCCTAGTTAAAAAAATATTGCTTATATATGTGGCTTGACAAATG   1274
M29           ATATTAAAATAGAATATCTAAATCTGTATCGGATTCTCAATTGTTTACCCTAGTTAAAAAAATATTGCTTATATATGTGGCTTGACAAATG   1274

Yuzhi 11      TACTTATAATTAATTAATCACATAAACATTTGACAAACAATGAGCCCTCTTTCAAAAACACCTGACAAGTGCAGTTAACTAATGAATTACG   1365
Yuzhi_Ds899   TACTTATAATTAATTAATCACATAAACATTTGACAAACAATGAGCCCTCTTTCAAAAACACCTGACAAGTGCAGTTAACTAATGAATTACG   1365
M10           TACTTTTAATTAATTAATCACATAAACATTTGACAAACAATGAGCCCTCTTTCAAAAACACCTGACAAGTGCAGTTAACTAATGAATTACG   1365
M11           TACTTTTAATTAATTAATCACATAAACATTTGACAAACAATGAGCCCTCTTTCAAAAACACTTGACAAGTGCAGTTAACTAATGAATTACG   1365
M18           TACTTTTAATTAATTAATCACATAAACATTTGACAAACAATGAGCCCTCTTTCAAAAACACTTGACAAGTGCAGTTAACTAATGAATTACG   1365
M19           TACTTTTAATTAATTAATCACATAAACATTTGACAAACAATGAGCCCTCTTTCAAAAACACTTGACAAGTGCAGTTAACTAATGAATTACG   1365
M29           TACTTTTAATTAATTAATCACATAAACATTTGACAAACAATGAGCCCTCTTTCAAAAACACTTGACAAGTGCAGTTAACTAATGAATTACG   1365

Yuzhi 11      TTGTTTTTTATTTTACATATATGTACTAAATCTACTGACAAACATAAGTTGATTTGACGAACTCACAGGATAGTCACAGATATCCCTGGCA   1456
Yuzhi_Ds899   TTGTTTTTTATTTTACATATATGTACTAAATCTACTGACAAACATAAGTTGATTTGACGAACTCACAGGATAGTCACAGATATCCCTGGCA   1456
M10           TTGTTTTTTATTTTACATATATGTACTAAATCTACTGATAAACATAAGTTGATTTGACGAACTCACAGGATAGTCACAGATATCCCTGGCA   1456
M11           TTGTTTTTTATTTTACATATATGTACTAAATCTACTGATAAACATAAGTTGATTTGACGAACTCACAGGATAGTCACAGATATCCCTGGCA   1456
M18           TTGTTTTTTATTTTACATATATGTACTAAATCTACTGATAAACATAAGTTGATTTGACGAACTCACAGGATAGTCACAGATATCCCTGGCA   1456
M19           TTGTTTTTTATTTTACATATATGTACTAAATCTACTGATAAACATAAGTTGATTTGACGAACTCACAGGATAGTCACAGATATCCCTGGCA   1456
M29           TTGTTTTTTATTTTACATATATGTACTAAATCTACTGATAAACATAAGTTGATTTGACGAACTCACAGGATAGTCACAGATATCCCTGGCA   1456

Yuzhi 11      CCACAGATTCCTCATTCGGTATGAATAAAGATCATATATACTCCATTTAATTAATATCTTATTTAAATCTTTGTTTTCACATTAATTAATT   1547
Yuzhi_Ds899   CCACAGATTCCTCATTCGGTATGAATAAAGATCATATATACTCCATTTAATTAATATCTTATTTAAATCTTTGTTTTCACATTAATTAATT   1547
M10           CCACAGATTCCTCATTCGGTATGAATAAAGATCATATATACTCCATTTAATTAATATCTTATTTAAATCTTTGTTTTCACATTAATTAATT   1547
M11           CCACAGATTCCTCATTCGGTATGAATAAAGATCATATATACTCCATTTAATTAATATCTTATTTAATTCTTTGTTTTCACATTAATTAATT   1547
M18           CCACAGATTCCTCATTCGGTATGAATAAAGATCATATATACTCCATTTAATTAATATCTTATTTAATTCTTTGTTTTCACATTAATTAATT   1547
M19           CCACAGATTCCTCATTCGGTATGAATAAAGATCATATATACTCCATTTAATTAATATCTTATTTAATTCTTTGTTTTCACATTAATTAATT   1547
M29           CCACAGATTCCTCATTCGGTATGAATAAAGATCATATATACTCCATTTAATTAATATCTTATTTAATTCTTTGTTTTCACATTAATTAATT   1547

Yuzhi 11      AATTTCTTGTTTACATGTAATTATCAATGTACTGCAGGAAAAGAAGTAGTGAGCTACGAGATGCCGAGGCCAAACATAGGAATACACAGG   1638
Yuzhi_Ds899   AATTTCTTGTTTACATGTAATTATCAATGTACTGCAGGAAAAGAAGTAGTGAGCTACGAGATGCCGAGGCCAAACATAGGAATACACAGG   1638
M10           AATTTCTTGTTTACATGTAATTATCAATGTACTGCAGGAAAAGAAGTAGTGAGCTACGAGATGCCGAGGCCAAACATAGGAATACACAGG   1638
M11           AATTTCTTGTTTACATGTAATTATCAATGTACTGCAGGAAAAGAAGTAGTGAGCTACGAGATGCCGAGGCCAAACATAGGAATACACAGG   1638
M18           AATTTCTTGTTTACATGTAATTATCAATGTACTGCAGGAAAAGAAGTAGTGAGCTACGAGATGCCGAGGCCAAACATAGGAATACACAGG   1638
M19           AATTTCTTGTTTACATGTAATTATCAATGTACTGCAGGAAAAGAAGTAGTGAGCTACGAGATGCCGAGGCCAAACATAGGAATACACAGG   1638
M29           AATTTCTTGTTTACATGTAATTATCAATGTACTGCAGGAAAAGAAGTAGTGAGCTACGAGATGCCGAGGCCAAACATAGGAATACACAGG   1638

Yuzhi 11      TTTGCATTTGTGCTGTTCAAGCAGAAGAAGAGACAATTAGGATGCGTGAGGGCACCAGCTTGTAGGGATGGATTCAATACCAGGAATTTTG   1729
Yuzhi_Ds899   TTTGCATTTGTGCTGTTCAAGCAGAAGAAGAGACAATTAGGATGCGTGAGGGCACCAGCTTGTAGGGATGGATTCAATACCAGGAATTTTG   1729
M10           TTTGCATTTGTGCTGTTCAAGCAGAAGAAGAGACAATTAGGATGCGTGAGGGCACCAGCTTGTAGGGATGGATTCAATACAAGGAATTTTG   1729
M11           TTTGCATTTGTGCTGTTCAAGCAGAAGAAGAGACAATTAGGATGCGTGAGGGCACCAGCTTGTAGGGATGGATTCAATACAAGGAATTTTG   1729
M18           TTTGCATTTGTGCTGTTCAAGCAGAAGAAGAGACAATTAGGATGCGTGAGGGCACCAGCTTGTAGGGATGGATTCAATACAAGGAATTTTG   1729
M19           TTTGCATTTGTGCTGTTCAAGCAGAAGAAGAGACAATTAGGATGCGTGAGGGCACCAGCTTGTAGGGATGGATTCAATACAAGGAATTTTG   1729
M29           TTTGCATTTGTGCTGTTCAAGCAGAAGAAGAGACAATTAGGATGCGTGAGGGCACCAGCTTGTAGGGATGGATTCAATACAAGGAATTTTG   1729

Yuzhi 11      CCCAGGAAAATGAGTTAGGTCTCCCTGTTGCTGCTGTTTACTTCAATTGCCAGAGAGAGACTGCTGCTAGAAGGCGCTAG   1809
Yuzhi_Ds899   CCCAGGAAAATGAGTTAGGTCTCCCTGTTGCTGCTGTTTACTTCAATTGCCAGAGAGAGACTGCTGCTAGAAGGCGCTAG   1809
M10           CCCAGGAAAATGAGTTAGGTCTCCCTGTTGCTGCTGTTTACTTCAATTGCCAGAGAGAGACTGCTGCTAGAAGGCGCTAG   1809
M11           CCCAGGAAAATGAGTTAGGTCTCCCTGTTGCTGCTGTTTACTTCAATTGCCAGAGAGAGACTGCTGCTAGAAGGCGCTAG   1809
M18           CCCAGGAAAATGAGTTAGGTCTCCCTGTTGCTGCTGTTTACTTCAATTGCCAGAGAGAGACTGCTGCTAGAAGGCGCTAG   1809
M19           CCCAGGAAAATGAGTTAGGTCTCCCTGTTGCTGCTGTTTACTTCAATTGCCAGAGAGAGACTGCTGCTAGAAGGCGCTAG   1809
M29           CCCAGGAAAATGAGTTAGGTCTCCCTGTTGCTGCTGTTTACTTCAATTGCCAGAGAGAGACTGCTGCTAGAAGGCGCTAG   1809
```

Supplementary Fig. S5. Diversity analysis of *SiDt* among the sesame germplasm resources. Thirty

core sesame germplasm are applied for the diversity analysis. A total of 18 SNP sites are detected in *SiDt* sequences among 5 accessions, i.e., M10, M11, M18, M19 and M29 as listed in Supplementary Table S10 online. Asterisks above the nucleotide sequences indicate the SNP sites of *SiDt* (*Sidt*) in Yuzhi 11, YuzhiDS899 and the 30 germplasm. The arrowhead indicates the SNP G397A site between Yuzhi 11 and Yuzhi DS899. The SNPs presented in more than 2 accessions are shaded in black. The SNPs presented in one specific accession are shaded in grey. Bases in frame are exon sequences of *SiDt*.
