# Supplementary material for: Ultra-dense SNP genetic map construction and identification of SiDt gene controlling the determinate growth habit in Sesamum indicum L
Source: Sci Rep. 2016 Aug 16;6:31556. doi: 10.1038/srep31556 (PMC4985745; doi:10.1038/srep31556)
Supplement: Supplementary Table S4 [file srep31556-s6.doc]

**Supplementary Table S4 Characteristics of the three phenotypes of growth habit in sesame**

| **Growth habit type** | **Sample name** | **Agronomic traits ^a^** | | | | **Budding period under photoperiodic treatments ^b^ (d)** | |
| --- | --- | --- | --- | --- | --- | --- | --- |
|  |  | **Branching pattern** | **Average plant height (cm)** | **Flowering duration on one stem (d)** | **Capsule node number on one stem** | **SD (12h light/**  **12h dark)** | **LD (15h light/**  **9h dark)** |
| Determinate 1 (*dt1*) | Yuzhi DS899 | Non branching | 125.7 | 18-26 | 8-20 | 39 | 63 |
| Determinate 2 (*dt2*) | 08TP092 | Top branching | 86.7 | 4-5 | 2-3 | 39 | 58 |
| Indeterminate (*Dt*) | JS012 | Top branching | - | >30 | >20 | - | - |
|  | Ningbohei | Non branching | - | >30 | >20 | - | - |
|  | Yuzhi 11 | Non branching | 165.0 | >30 | >20 | 40 | >70 |

**a** The determinacy growth habit trait of the five accessions are investigated at Sanya (109°50' E and 18°25' N), Pingyu (113°62' E and 32°97' N) and Yuanyang (113°97' E and 35°05' N) experimental stations in 2013. The average height trait data listed in the table are investigated at Yuanyang experimental station in 2013.

**b** Photoperiodic treatments are performed in growing chamber under the day/night temperature of 28°C /24°C with 70±1% relative humidity.

‘-’ indicates not done.
