# Supplementary material for: Ultra-dense SNP genetic map construction and identification of SiDt gene controlling the determinate growth habit in Sesamum indicum L
Source: Sci Rep. 2016 Aug 16;6:31556. doi: 10.1038/srep31556 (PMC4985745; doi:10.1038/srep31556)
Supplement: Supplementary Table S5 [file srep31556-s7.doc]

**Supplementary Table S5 Location of QDt1 associated with the determinate growth habit in sesame using winQTLCart and QTLNetwork**

| **Locus name** | **Linkage group no.** | **winQTLCart** | | | **QTLNetwork** | |
| --- | --- | --- | --- | --- | --- | --- |
|  |  | **Interval (cM)** | **V_G_/V_P_ (%)** | **LOD** | **Interval (cM)** | **P vaule** |
| QDt1 | 8 | 16.7-22.2 | 70.2 | 18.23 | 18.0-19.2 | <1E-6 |
